# *Acanthopanax senticosus* extract alleviates radiation‐induced learning and memory impairment based on neurotransmitter‐gut microbiota communication

**DOI:** 10.1111/cns.14134

**Published:** 2023-03-27

**Authors:** Chen Song, Yishu Yin, Yue Qin, Tianzhu Li, Deyong Zeng, Ting Ju, Fangyuan Duan, Yingchun Zhang, Weihong Lu

**Affiliations:** ^1^ School of Medicine and Health Harbin Institute of Technology 150001 Harbin China; ^2^ School of Chemistry and Chemical Engineering Harbin Institute of Technology 150001 Harbin China; ^3^ National and Local Joint Engineering Laboratory for Synthesis, Transformation and Separation of Extreme Environmental Nutrients Harbin Institute of Technology 150001 Harbin China; ^4^ ZhenBaoDao Pharmaceutical Co., Ltd 150040 Harbin China

**Keywords:** *Acanthopanax senticosus* extract, gut microbiota, learning and memory impairment, neurotransmitters, radiation

## Abstract

**Background:**

*Acanthopanax senticosus* (*AS*) is a medicinal and food plant with many physiological functions, especially nerve protection. Its extract has many functional components, including polysaccharides, flavonoids, saponins, and amino acids. Our previous study indicated that *AS* extract protected against nerve damage caused by radiation. However, little is known about the gut‐brain axis mechanism of AS and its impact on radiation‐induced learning and memory impairment.

**Method:**

In ^60^Co‐γ ray‐irradiated mice, we investigated the changes in behavior, neurotransmitters and gut microbiota after different days of administration of *AS* extract as a dietary supplement.

**Results:**

The *AS* extract improved learning and memory ability in mice, and the neurotransmitter levels in the hippocampus and colon started to change from the 7th day, which accompanied changes of the gut microbiota, a decreased abundance of *Helicobacter* on the 7th day and an increased abundance of *Lactobacillus* on the 28th day. Among the marker bacteria, *Ruminococcus* and *Clostridiales* were associated with 5‐HT synthesis, and Streptococcus were associated with 5‐HT and ACH synthesis. In addition, the *AS* extract increased the tight junction protein, inhibited inflammation levels in colon, and even increased the relative protein expression of BDNF and NF‐κB and decreased the relative protein expression of IκBα in the hippocampus of irradiated mice.

**Conclusion:**

These results will lay the foundation for further study on the mechanism of the gut‐brain axis of *AS* in preventing radiation‐induced learning and memory impairment.

## Graphical abstract

*Acanthopanax senticosus* extract alleviates radiation‐induced learning and memory impairment based on neurotransmitter‐gut microbiota communication. By Figdraw (www.figdraw.com).

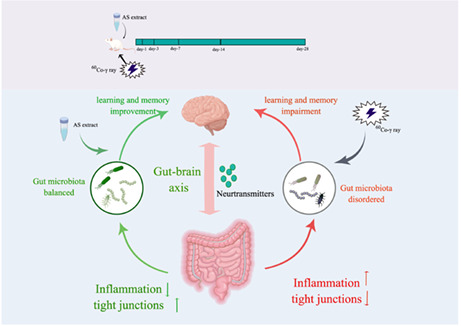

## 1 INTRODUCTION

With the development of science and technology, radiation widely exists in our life. Space radiation, nuclear pollution as well as radiation pollution, all can induce negative effects on the organism, including the endocrine system, circulatory system, and reproductive system, and especially serious damage to the nervous system.^1^, ^2^ For instance, radiation can cause swelling and necrosis of glial cells, cerebrovascular damage, oxidative stress response, inflammatory cell infiltration, and destruction of the blood–brain barrier, resulting in brain tissue dysfunction.^3^, ^4^ As we know, the brain is the center of all human life activities, which include not only basic neural activities such as breathing and digestion, but also the material basis for all thinking activities. It has the highest control ability in learning and memory, cognitive understanding, judgment, and reasoning. Thus, it's crucial to protect our brains from radiation. However, some antiradiation agents are difficult to popularize due to their high side effects and high price. Therefore, exploring antiradiation drugs with high efficiency and low toxicity is in an urge.

Many homologous ingredients of medicine and food, such as polysaccharides, flavonoids, saponins, peptides, alkaloids, and vitamins, have been found to have antiradiation activities.^5^, ^6^, ^7^ As a medicinal and food homologous plant, *Acanthopanax senticosus* (*AS*) is widely grown in China, and is also often used as a wild vegetable. Previous studies have confirmed the ability of *AS* to improve learning and memory, increase the number of neurons, prevent brain damage, and have good therapeutic effects on central degenerative diseases and cerebrovascular diseases.^8^, ^9^, ^10^ However, its mechanism of protecting the nervous system, especially from radiation‐induced brain injury, needs to be further explored. In addition, many studies have shown that gut microbes and their metabolites are extensively involved in the communication between the brain and the gut in recent years.^11^, ^12^ Various studies have found that gut microbiota can interact with the brain through the gut‐brain axis (GBA) pathway.^13^, ^14^ Many factors have been shown to have a modulating effect on both the brain and microbiota, including host diet, congenital factors, environmental factors, exercise and mood, medications, and mode of delivery.^13^, ^15^, ^16^, ^17^ There are many pathways of potential communication between the gut microbiota and the brain, including hepatic and gallbladder metabolism, immune‐modulatory responses, neuronal innervation, enteroendocrine, and microbial metabolite signaling.^18^

There is very little published research on the gut‐brain axis mechanism of the protective effect of Acanthopanax senticosus on radiation‐induced brain injury. In this study, we explored the changes in neurotransmitters and gut microbiota in irradiated mice treated with *AS* extract as a dietary supplement from the perspectives of behavior and neurotransmitters. This will lay the foundation for further study on the mechanism of the brain‐gut axis of *Acanthopanax senticosus* in preventing radiation‐induced brain injury.

## 2 METHODS

### 2.1 Study design and materials

#### 2.1.1 Experimental grouping

Adult male KM mice, 6–8 weeks old, weighing 20 ± 2 g, were obtained from the Animal Experimental Center of the 2nd Affiliated Hospital of Harbin Medical University (Harbin, Heilongjiang, China); the certification number was SYXK (HEI) 2019‐001. All of the experimental animal procedures were evaluated and approved by the local ethics committee of Harbin Institute of Technology (IACUC‐2021075). All animals were maintained in an environmentally controlled breeding room with a regular 12‐h light cycle at 22 ± 2°C. After a normal diet for 1 week, the animals were divided randomly into 7 groups: the control group, the model group, the *AS*_1d group, the *AS*_3d group, the *AS*_7d group, the *AS*_14d group, the *AS*_28d group. The recommended dose of commercially available *Acanthopanax* tablets is 1–1.5 g. Based on the Chinese Pharmacopeia standard, literature review, and the dose screening of Acanthopanax senticosus extracts in our previous study, the mice of *AS* groups were administrated with *AS* extract for 1 day, 3 days, 7 days, 14 days and 28 days, respectively at a dose of 250 mg/kg/d. We selected the dose of 250 mg/kg/d, which is equivalent to a human dose of 23 mg/kg/d according to the body surface area conversion method, and it is also equivalent to 1.38 g/d for an adult weighing 60 kg. The control group and model group were administrated with saline. After the intervention of *AS* extract, the mice of *AS* group and model group were exposed to ^60^Co‐γ ray irradiation at a total dose of 4 Gy (1 Gy/min).

#### 2.1.2 Plant materials

*AS* extract is provided by ZhenBaoDao Pharmaceutical Co., Ltd (202,201,161, China, Harbin). It is an orange‐yellow clear liquid without fat, protein and ash.

### 2.2 Analysis of the main components of *Acanthopanax senticosus* extract

#### 2.2.1 Analysis of *AS* extract by HPLC

The determination of the main compounds in the extracts of Acanthopanax was carried out with minor modifications according to the method described in the Chinese Pharmacopeia. 200 mg of AS extract was dissolved in 50% methanol, sonicate for 10 min, and fix the volume to 25 mL. 1 mL of AS extract was filtered using a 0.45 μm polyvinylidene fluoride (PVDF) syringe filter for HPLC analysis.

The separation of *AS* extract was performed on the UltiMate 3000 Basic HPLC Systems (Thermo, USA) equipped with Zorbax SB‐C18 column (4.6 mm × 150 mm, 5 μm, Agilent, USA) at 30°C at a flow rate of 0.8 mL/min. Acetonitrile (A) and 0.1% phosphoric acid solution (B) were used as mobile phases. The gradient of mobile phase A was set as follows: 10%–20%(0–20 min), 20%–25% (20–30 min), 30%–40%.

Standard curves were developed for the quantification of the five main active ingredients in the AS extract, protocatechuic acid, syringin, chlorogenic acid, eleutheroside E, isofraxidin using the above method.

#### 2.2.2 Analysis of *AS* extract UPLC‐Q‐TOF‐MS/MS

Mass analysis was performed on Q‐TOF analyzer in TripleTOF 5600‐1 (AB Sciex, USA) in positive ion and negative ion mode, using the following parameters: Ion source voltage, 5500 V(positive ion) and 4500 V (negative ion); source temperature, 550°C; DP: 60 V(positive ion) and −60 V (negative ion); CE: 10 eV(positive ion) and −10 eV (negative ion); CES: 15 eV; GS1and GS2 60 psi; CUR: 35 psi. Primary mass spectrometry parent ion scan range of *m/z* 50–1700, IDA set the 12 highest peaks with response values above 100 cps for secondary mass spectrometry, daughter ion scan range of *m/z* 25–1200, dynamic background subtraction (DBS) enabled.

#### 2.2.3 Determination of polysaccharide content in *AS* extract

The polysaccharides were extracted according to our previous study and glucose was used as a standard for the determination by phenol‐sulfuric acid method.^19^ Briefly, the polysaccharide extracts were dissolved in distilled water, then 5% phenol and concentrated sulfuric acid were added, respectively. After mixing, the mixture was allowed to stand at room temperature for 30 min and the absorbance values were measured at 490 nm using a spectrophotometer.

#### 2.2.4 Determination of flavonoids content in *AS* extract

The content of flavonoids in AS was determined by aluminum nitrate colorimetry, and rutin was used as the standard. Briefly, 0.3 mL of 5% sodium nitrite solution was added to 1 mL of the sample, and left for 6 min, then 0.3 mL of 10% aluminum nitrate solution was added, and after standing for 6 min, 4 mL of 1 mol/L sodium hydroxide solution was added, and finally diluted to 10 mL with 30% ethanol, shaken well, and left to stand for 10 min. The OD value of the sample was measured by an ultraviolet spectrophotometer at a wavelength of 510 nm.

### 2.3 Behavioral test

#### 2.3.1 Water maze

The spatial learning and memory performance of mice was tested by the water maze. Briefly, the rectangular water maze contained many blind zones and an exit platform. In the experiment, we recorded the time of the mice reaching the platform at the exit and the time of mice hitting the maze wall. Before the formal test, all the mice were trained for 5 days, and then tested on the sixth day. The platform arrival time and the number of times to blind zones were recorded.

#### 2.3.2 Sucrose preference test

The sensitivity of the nervous system of mice was tested by the sucrose preference test. Before the experiment, the mice were fed two bottles of sucrose water for 24 h, and one bottle of sucrose water was replaced with distilled water for the second 24 h. Then, after deprivation of water for 24 h, the mice were given distilled water and sucrose water for 4 h, and the position of the bottle was changed during the experiment to avoid the influence of memory. Sucrose partiality was defined as the ratio of the volume of sucrose vs. water consumed during the test and using the equation: Sucrose preference % = (*V*sucrose/*V*sucrose + *V*water) × 100%, where *V*sucrose is the volume of sucrose consumption, and *V*sucrose + *V*water is summation volume of sucrose consumption and water consumption.

### 2.4 Hematoxylin and eosin staining

Hippocampus tissues were fixed in 4% poly formaldehyde for 4–6 h. After fixation, the tissues were dehydrated and embedded in paraffin. Tissues were cut at a thickness of 4 μm on a microtome and stained with Hematoxylin & Eosin (HE). The morphology was observed under the microscope (Eclipse Ci‐L, Nikon, Japan).

### 2.5 Elisa

In the neurotransmitter assay, the hippocampus and colon were homogenized with saline and the supernatant was centrifuged, followed by the commercial kit instructions. For the inflammatory cytokine assay in serum, whole blood was collected and the serum was centrifuged for the assay.

The levels of γ‐aminobutyric acid (GABA), serotonin (5‐HT), norepinephrine (NE) and acetylcholine (ACH) in the brain and colon were measured using commercially available ELISA kits (Shanghai Lengton Bioscience Co., LTD, Shanghai, China). The specific operation followed the protocol of the kits. The level of interleukin‐1β (IL‐1β), interleukin‐6 (IL‐6), and tumor necrosis factor‐α (TNF‐α) in serum were detected by commercially available ELISA kits (QuanZhou Kenuodi Bioscience Co., LTD, QuanZhou, China).

### 2.6 RNA preparation and real‐time qPCR analysis

The total RNA of the colon was isolated using TaKaRa MiniBEST Universal RNA Extraction Kit (TaKaRa; Dalian, China). gDNA Eraser Spin Column was used to purify the RNA. RNA quantification was then performed using an ultra‐micro UV spectrophotometer (IMPLEN,German). cDNA was synthesized by PrimeScript™ RT reagent Kit (Perfect Real Time) (TaKaRa; Dalian, China) according to the manufacturer's instructions. qPCR assays were performed in each 25 μL reaction containing cDNA corresponding to 100 ng of total RNA and gene‐specific primers (Table 1) supplied by Ruibiotech (Ruibiotech Ltd., Beijing, China) and using TB Green® Premix Ex Taq™ II (Tli RNaseH Plus) (TaKaRa; Dalian, China). Reactions were performed by ABI QuanrStudio5 Real‐time PCR Detection System (Life Technologies, Singapore) with an initial denaturation at 95°C for 30 s, followed by 40 cycles of 95°C for 5 s, 60°C for 30 s. All samples were analyzed in duplicate and the results are expressed as a relative value after normalization to β‐actin mRNA. The PCR primer sequences were showed in Table 1.

**TABLE 1:** The PCR primer sequences.

Genes	Primer sequences (5′‐3′)
*β‐actin F*	ATCACTATTGGCAACGAGCGGTTC
*β‐Actin R*	CAGCACTGTGTTGGCATAGAGGTC
*TNF‐α F*	GTCCGGGCAGGTCTACTTTG
*TNF‐α R*	GGGGCTCTGAGGAGTAGACA
*IL‐1β F*	TGCCACCTTTTGACAGTGATG
*IL‐1β R*	ATGTGCTGCTGCGAGATTTG
*IL‐6 F*	TGATGGATGCTACCAAACTGGA
*IL‐6 R*	TGTGACTCCAGCTTATCTCTTGG
*Occludin F*	GTCCTCCTGGCTCAGTTGAA
*Occludin R*	AGAGTACGCTGGCTGAGAGA
*Claudin F*	ACTGCGTACAAGACGAGACG
*Claudin R*	TCCCTGATGATGGTGTTGGC
*ZO‐1F*	TCTTGCAAAGTATCCCTTCTGT
*ZO‐1R*	GAAATCGTGCTGATGTGCCA

### 2.7 Analysis of gut microbiota composition

The colon contents of mice were taken under sterile conditions and quickly frozen in liquid nitrogen for later use. Total genomic DNA was extracted using DNA Extraction Kit following the manufacturer's instructions (DNeasy PowerSoil Kit, QIAGEN). The diluted DNA was used as the template for PCR amplification of bacterial 16S rRNA genes with the barcoded primers and Takara Ex Taq (Takara). For bacterial diversity analysis, V3–V4 (or V4–V5) variable regions of 16S rRNA genes were amplified with universal primers F: 343F 5′‐AGGGTATCTAATCCT‐3′, 798R 5′‐TACGGRAGGCAGCAG‐3′. Amplicon quality was visualized using gel electrophoresis, purified with AMPure XP beads (Agencourt), and amplified for another round of PCR. After purifying with the AMPure XP beads again, the final amplicon was quantified using the Qubit dsDNA assay kit. Equal amounts of purified amplicon were pooled for subsequent sequencing. Clean reads were subjected to primer sequences removal and clustering to generate operational taxonomic units (OTUs) using search software with 97% similarity cut off. The representative read of each OTU was selected using QIIME package. All representative reads were annotated and blasted against Silva database Version 123 using RDP classifier (confidence threshold was 70%).^20^

### 2.8 Western blot

Total protein was isolated from the brain tissues using the protein extraction kit (Solarbio, Beijing, China)and adjusted to the same final concentration according to the BCA Assay Kit (Beyotime Institute of Biotechnology, Shanghai, China). SDS‐PAGE was used to separate the samples, which were then transferred to NC (nitrocellulose filter) membranes. After blocking with 2% BSA for 60 min, the NC membranes were incubated overnight with primary antibodies at 4°C. The following day, after washing three times with PBST(phosphate buffered saline tween‐20), the membranes were incubated with the secondary antibodies (Goat Anti‐Mouse IgG(H + L), Alkaline Phosphatase conjugate and Goat Anti‐Rabbit IgG (H + L), Alkaline Phosphatase‐conjugated, Proteintech, China) for 1 h at room temperature. The membranes were washed three times in PBST buffer and incubated with a commercial western immunoblotting detection reagent (western blue stabilized substrate for alkaline phosphatase, Promega, USA) for 5 min. The resulting immunoreactive bands were exposed by Amersham Imager 600 (GE). The primary antibodies used in this study were BDNF (Abcam, ab108319, 1:1500), and GAPDH (Proteintech, 60,004‐1‐Ig, 1:2000).

### 2.9 Statistical analysis

Statistical analyses were performed using GraphPad Prism version 8.0. All values were presented as means ± SEM. Shapiro–Wilk test was used to test normal distribution. One‐way ANOVA (Analysis of Variance) followed by a multiple‐comparison test (Tukey's) was performed for normally distributed continuous data. The Kruskal–Wallis test was used when the variances were not homogeneous. *p* < 0.05 was considered to be significant. We assessed correlations using Spearman's correlation coefficient.

## 3 RESULTS

### 3.1 The main chemical compositions of *Acanthopanax senticosus* extract

**FIGURE 1:**
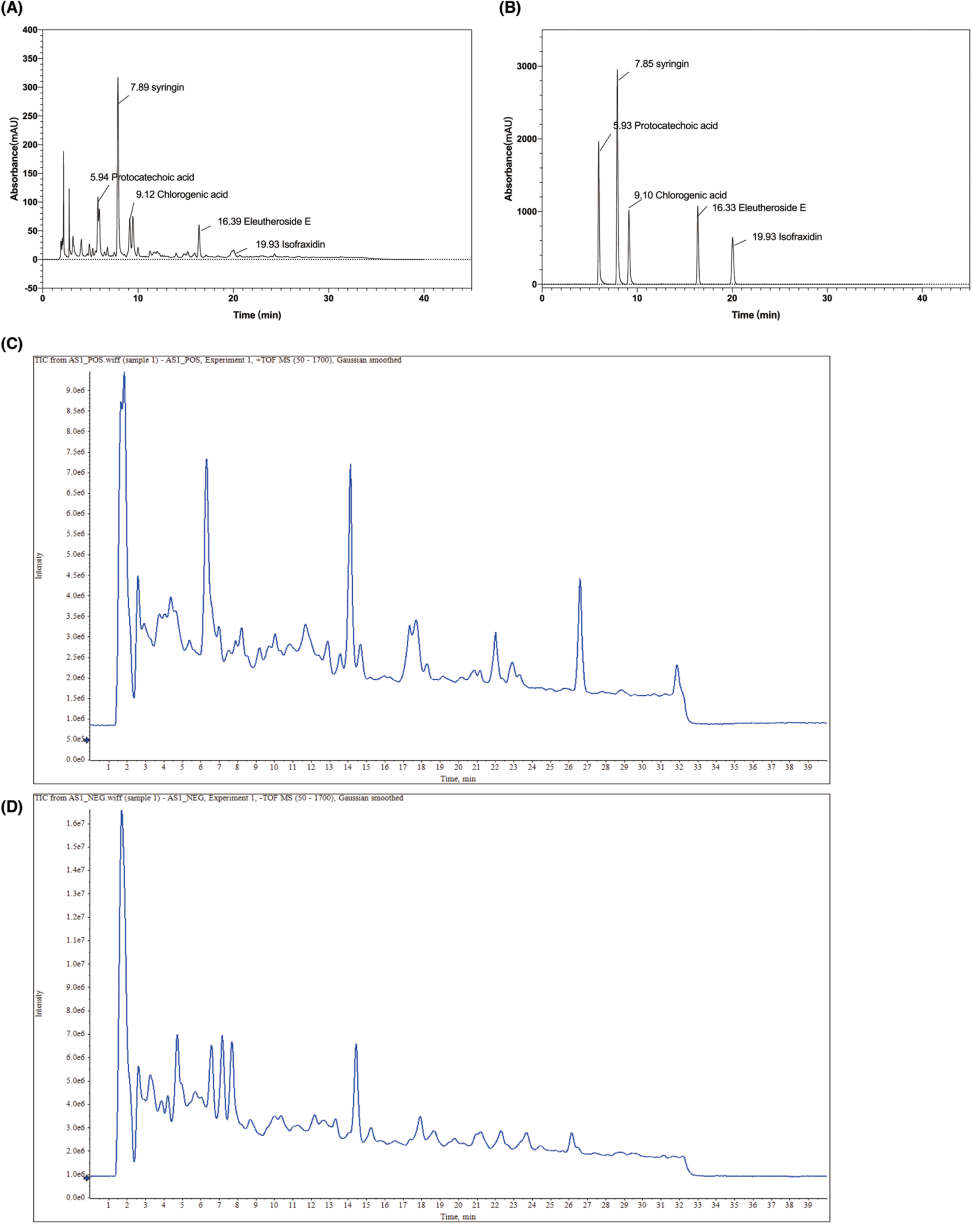
HPLC chromatogram and total ion chromatogram of Acanthopanax senticosus extract. (A) HPLC chromatogram of *AS* extract; (B) Chromatogram of the standard compounds; (C) Positive ion chromatogram; (D) Negative ion chromatogram.

The results obtained by HPLC and UPLC‐Q‐TOF‐MS/MS analysis of the main functional compositions in the extracts of *Acanthopanax senticosus* used in this study were shown in Figure 1. The contents of each main constituent were shown in Table 2. The main functional components of AS extract was protocatechuic acid (0.690 ± 0.005%), syringin (1.970 ± 0.007%), chlorogenic acid (0.587 ± 0.019%), eleutheroside E (0.442 ± 0.004%), isofraxidin (0.180 ± 0.0002%).The polysaccharide was 6.780 ± 0.013%. The flavonoids 0.81 ± 0.041%.

**TABLE 2:** The main chemical compositions of *Acanthopanax senticosus* extract.

Compound name	Compound structure	Value (%)
Protocatechuic acid	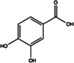	0.690 ± 0.005
Chlorogenic acid	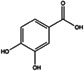	0.587 ± 0.019
Syringin	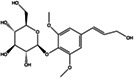	1.970 ± 0.007
Eleutheroside E	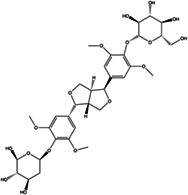	0.442 ± 0.004
Isofraxidin	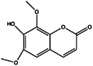	0.180 ± 0.0002

In addition, we also performed UPLC‐Q‐TOF‐MS/MS to analyze the components of AS extract, and the results are shown in Table 3. Based on the review of literature and comparison of ion fragments,^21^, ^22^, ^23^, ^24^ we resolved the components in the UPLC‐Q‐TOF‐MS/MS results of AS extract, including two amino acid components, three caffeoylquinic acid components, four coumarins, three lignans, two flavonoids, one phenolic glycoside component and one adenosine. As the results are shown in Table 3. The two amino acid components included isoleucine (*t* = 2.53 min, *m/z* = 132.10) and phenylalanine (*t* = 2.91 min, *m/z* = 166.08 [M + H]^+^). The three caffeic quinic acid components included chlorogenic acid (*t* = 4.71 min, *m/z* = 353.08 [M–H]^−^) and two mono‐caffeoylquinic acid isomers (*t* = 7.05 min and 7.55 min, *m/z* = 353.1 [M–H]^−^), whose cleavage pattern was followed by the loss of caffeoyl group, resulting in a fragment ion *m/z* = 191, and the presence of this fragment indicated the presence of quinic acid in the structure. The retention time of protocatechuic acid was 4.09 min, and *m/z* was 153.0178 [M–H]^−^. The four coumarin‐like components included 7‐hydroxycoumarin (*t* = 4.39 min, *m/z* = 163.03 [M + H]^+^), scopolin (*t* = 6.61 min, *m/z* = 355.10 [M + H]^+^), eleutheroside B2 (*t* = 7.61 min, *m/z* = 548.18 [M + NH4]^+^), and isofraxidin (*t* = 17.48 min, *m/z* = 223.05 [M + H]^+^). The three lignans included pinoresinol‐di‐O‐β‐d‐glucoside (*t* = 11.65 min, *m/z* = 700.27 [M + NH4]^+^), eleutheroside E (*t* = 14.13 min, *m/z* = 760.29 [M + H]^+^), and (+)‐syringaresinol‐O‐β‐d‐glucoside (*t* = 21.76 min, *m/z* = 598.24 [M + NH4]^+^). The ionic fragments of flavonoids usually contain a quercetin parent nucleus with *m/z* = 303. The flavonoid compounds resolved in this experiment were hypericin (*t* = 7.18 min, *m/z* = 465.13 [M + H]^+^) and quercetin (*t* = 7.62 min, *m/z* = 303.12 [M + H]^+^). One phenolic glycoside component was syringin (*t* = 6.22 min, *m/z* = 390.17 [M + NH4]^+^).

**TABLE 3:** UPLC‐Q‐TOF‐MS/MS analysis of *AS* extract.

No.	RT (min)	Compound name	Ionic mode	Detection value (*m/z*)	Molecule formula	Fragment ions (*m/z*)
1	1.84	Adenosine	M + H	286.10	C_10_H_13_N_5_O_4_	*m/z* 136.0623[M + H–C_5_H_8_O_4_]^+^
2	2.53	Isoleucine	M + H	132.10	C_6_H_13_NO_2_	*m/z* 86.0968 [M + H–HCOOH]^+^
3	2.91	Phenylalanine	M + H	166.08	C_9_H_11_NO_2_	*m/z* 91.0537, [C_7_H_7_]^+^, *m/z* 103.06542 [M + H–HCOOH–NH_3_]^+^, *m/z* 120.0808 [M + H–HCOOH]^+^
4	4.39	7‐hydroxycoumarin	M + H	163.03	C_9_H_6_O_3_	*m/z* 89.0389 [M + H–CO–H_2_O–CO]^+^, *m/z* 117.0342 [M + H–CO–H_2_O]^+^, *m/z* 135.0448 [M + H–CO]^+^, *m/z* 145.0295 [M + H–H_2_O]^+^
5	6.22	Syringin	M + NH_4_	390.17	C_17_H_24_O_9_	*m/z* 133.0641 [M–Glu–H_2_O–CH_3_OH–CO + H]^+^, *m/z* 161.0593 [M–Glu–H_2_O–CH_3_OH + H]^+^, *m/z* 193.0856 [M–Glu–H_2_O + H]^+^, *m/z* 211.0967 [M–Glu + H]^+^
6	6.61	Scopolin	M + H	355.10	C_16_H_18_O_9_	*m/z* 163 [M–Glc–OCH_3_ + H]^+^, *m/z* 135 [M–Glc–OCH_3_–CO + H]^+^
7	7.18	Hypericin	M + H	465.13	C_21_H_20_O_12_	*m/z* 303 [M–Gal+H]^+^
8	7.62	Quercetin	M + H	303.12	C_15_H_10_O_7_	*m/z* 137.0592 [M + H–C_8_H_6_O_4_]^+^
9	7.88	Eleutheroside B2	M + NH_4_	548.18	C_23_H_30_O_14_	*m/z* 223 [M–Glc–Rha + H]^+^
10	11.65	Pinoresinol‐di‐O‐β‐d‐glucoside	M + NH_4_	700.27	C_32_H_42_O_16_	*m/z* 359 [M–Glc–Glc + H]^+^, *m/z* 341 [M–Glc–Glc–H_2_O + H]^+^, *m/z* 235 [M–Glc–Glc–124 + H]^+^
11	14.13	Eleutheroside E	M + NH_4_	760.29	C_34_H_46_O_18_	*m/z* 401.157 [M–Glc–Glc–H_2_O + H]^+^, *m/z* 265.1075, *m/z* [M–Glc–Glc–154 + H]^+^
12	17.48	Isofraxidin	M + H	223.05	C_11_H_10_O_5_	*m/z* 191 [M–CO + H]^+^, *m/z* 163 [M–CO–CO + H]^+^
13	21.76	(+)‐Syringaresinol‐O‐β‐d‐glucoside	M + NH_4_	598.24	C_28_H_36_O_13_	*m/z* 401 [M–Glc–H_2_O + H]^+^, *m/z* 256 [M–Glc–152 + H]^+^
14	4.09	Protocatechuic acid	M–H	153.02	C_7_H_6_O_4_	*m/z* 109.03 [M–H–COO]^−^
15	4.71	Chlorogenic acid	M–H	353.08	C_16_H_18_O_9_	*m/z* 353.0812 [M–H]^−^, *m/z* 191.6309 [M–H‐caffeoyl]^−^, 179.0324 [M–H‐caffeoyl–H_2_O]^−^, *m/z* 135 [M–H–C_9_H_6_O_5_–H_2_O–CO2]^−^
16	7.05	Mono‐caffeoylquinic acid	M–H	353.10	C_16_H_18_O_9_	*m/z* 191.0552 [M–H‐caffeoyl]^−^
17	7.55	Mono‐caffeoylquinic acid	M–H	353.10	C_16_H_18_O_9_	*m/z* 191.0546 [M–H‐caffeoyl]^−^, *m/z* 179.0334 [M–H‐caffeic acid]^−^, *m/z* 173.0441 [M‐caffeyl–H_2_O–H]^−^

### 3.2 *Acanthopanax senticosus* extract prevents learning and memory impairment in irradiated mice

**FIGURE 2:**
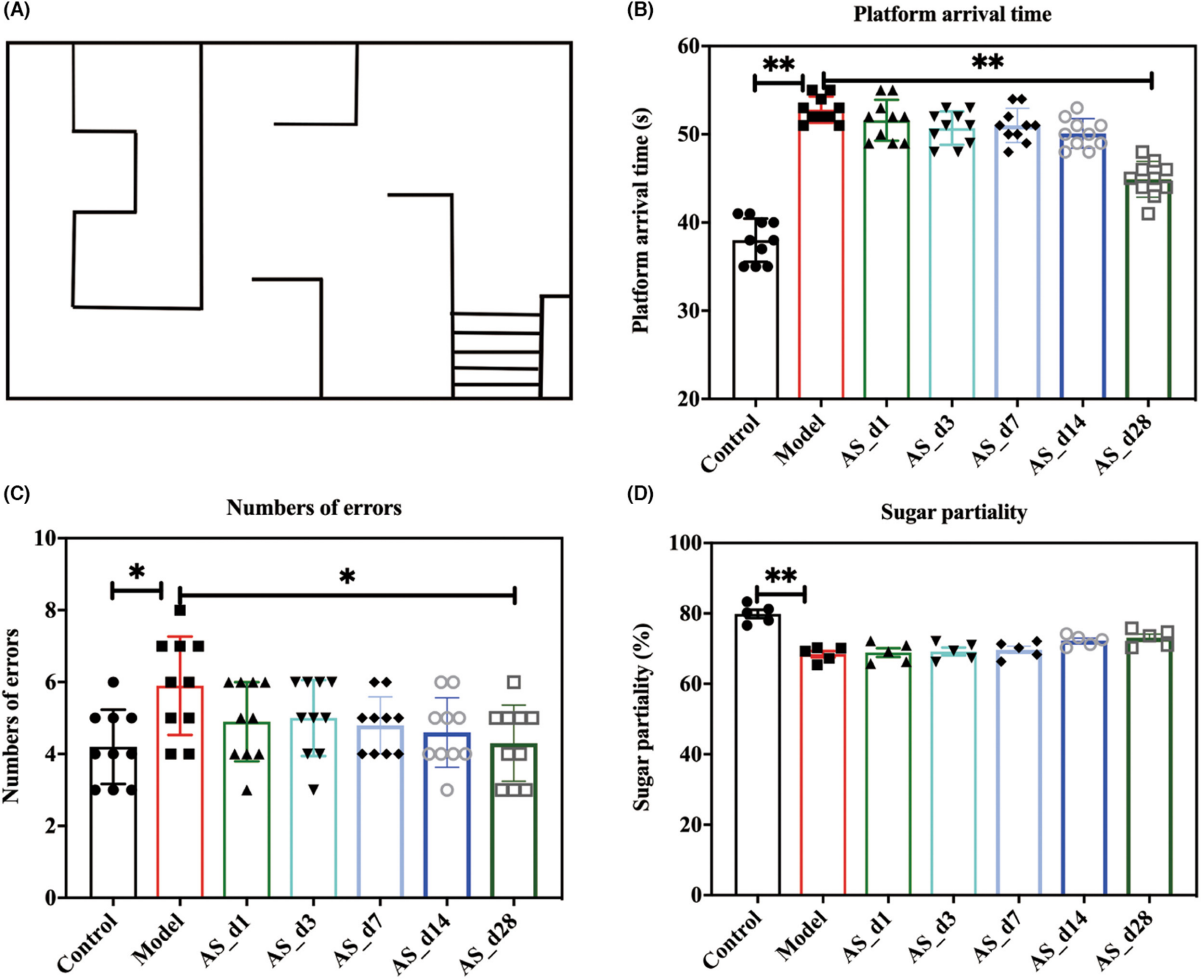
The effect of *AS* extracts on behavioral performance. (A) The water maze design. (B) the platform arrival time. (C) the number of times to blind zones. (D) the sugar preference. Values are expressed as the mean ± SEM (*n* = 10 or *n* = 5). Statistical analyses were conducted using the one‐way ANOVA followed by Tukey's pos hoc test, *p < 0.05, **p < 0.01).

A square water maze was used to investigate the learning and memory abilities of irradiated mice. The results, as shown in Figure 2, indicated that the supplementation of *AS* extract reduced the time of the platform arrival time and the number of blind spots, whereas its effect was significant 28 days after *AS* extract intervention (*p* < 0.05). This trend showed that the effect of *AS* extract on preventing learning and memory impairment in irradiated mice became more significant with the increase in intervention days.

Because rodents are extremely sensitive to sweet solutions, which serve as an effective reward for mice, sugar water preference is commonly used to assess the degree of hedonic loss. As shown in Figure 2D, radiation caused anhedonia in mice. *AS* extract prevented radiation‐induced anhedonia, and with the increase in intervention days, the preference for sugar water showed an increasing trend, but not significant (*p* > 0.05).

### 3.3 Effects of *Acanthopanax senticosus* extract on hippocampal neurons of irradiated mice

**FIGURE 3:**
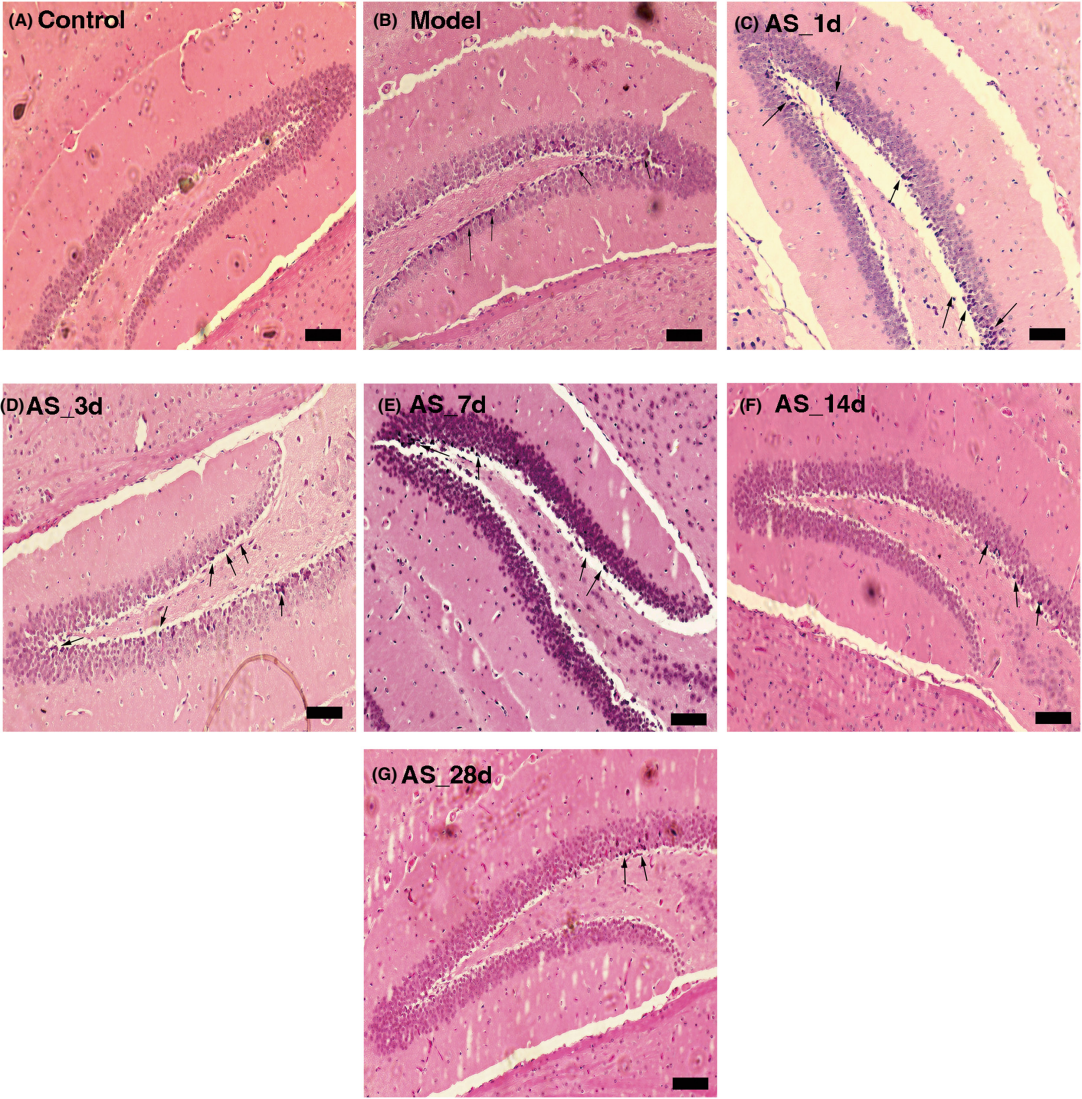
*AS* extract alleviated the brain injury of irradiated mice. (A) H&E staining of hippocampus in control group; (B) H&E staining of hippocampus in model group; (C) H&E staining of hippocampus in AS_1d group; (D) H&E staining of hippocampus in AS_3d group; (E) H&E staining of hippocampus in AS_7d group; (F) H&E staining of hippocampus in AS_14d group; (G) H&E staining of hippocampus in AS_28d group; (Scale bar = 100 μm).

The hippocampal brain region is a small yet major brain region located under the cerebral cortex and plays a crucial role in the consolidation of information from short‐term memory to long‐term memory and in spatial navigation. HE staining results showed abnormal morphology of hippocampal neurons after radiation, with reduced cell volume, nucleus pyknosis, disordered arrangement, and loose structure (Figure 3). The hippocampal structure of the 1, 3, and 7‐day intervention of *AS* groups were similar to that of the radiation group, the number of hippocampal pyramidal cells decreased, the arrangement was disordered, and the neuronal pyknosis increased compared with the control group. After 14 and 28 days of *AS* extract treatment, the hippocampal neurons of mice were more intact and less abnormal cells than those of the radiation group (Figure 3).

### 3.4 *Acanthopanax senticosus* extract supplementation alters neurotransmitter levels in the hippocampus of irradiated mice

**FIGURE 4:**
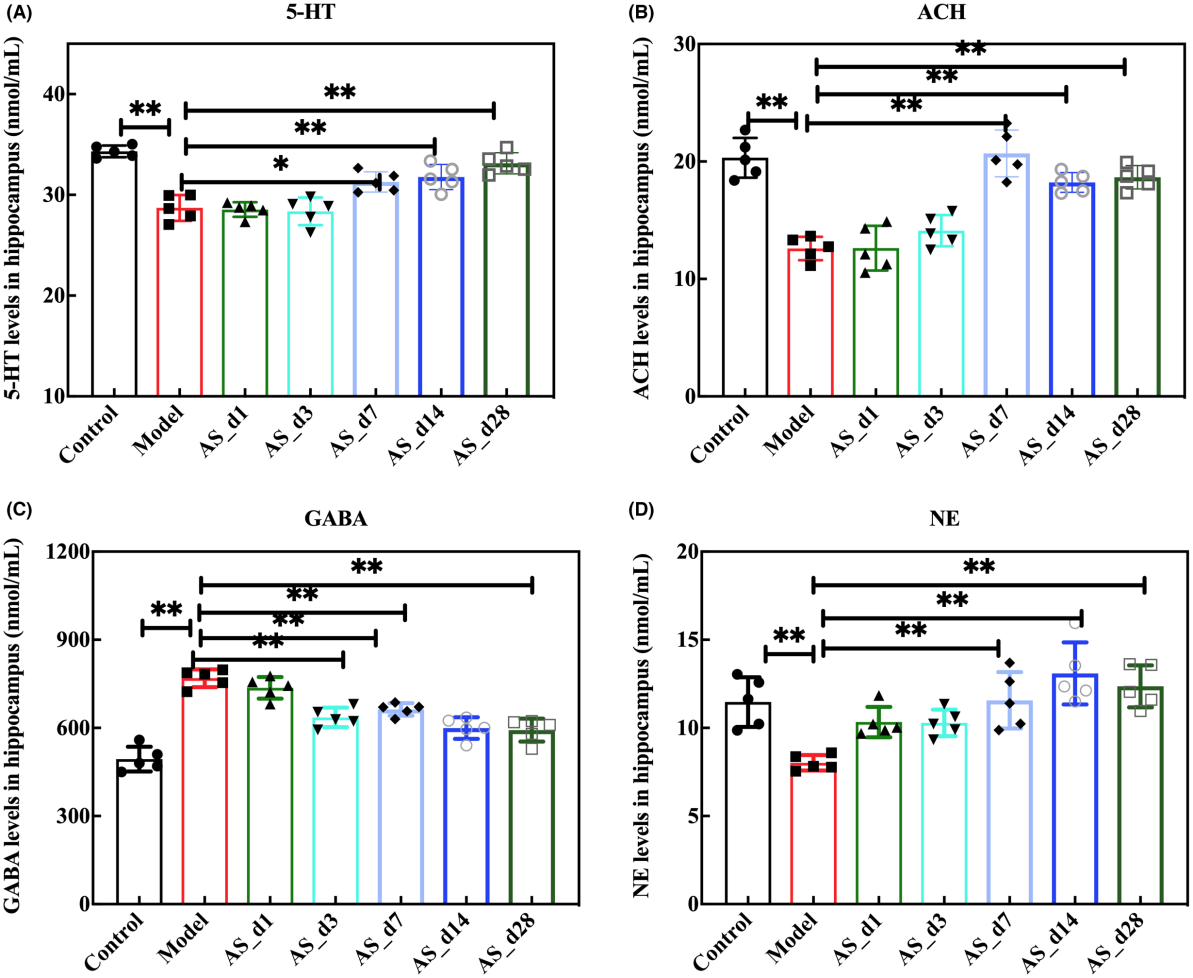
The supplementation of *AS* extract changed the levels of neurotransmitters in the hippocampus. (A) The level of 5‐HT; (B) The level of ACH; (C) The Level of GABA; (D) The level of NE; Values are expressed as the mean ± SEM (*n* = 5). Statistical analyses were conducted using the one‐way ANOVA followed by Tukey's pos hoc test, *p < 0.05, **p < 0.01).

The functional integrity of the hippocampus includes not only the structural integrity but also the interaction of various neurotransmitters. The neurotransmitters in the hippocampus include monoamine neurotransmitters, cholinergic neurotransmitters and amino acid neurotransmitters, the synthesis process and content of which are related to the learning and memory of animals. We investigated potential changes in concentrations of neurotransmitters across the seven groups. Compared with the model group, *AS* supplementation changed the levels of monoamine neurotransmitters in the hippocampus of irradiated mice, in which 5‐HT changed significantly after 28‐day intervention, and the NE changed significantly after 7‐day intervention (*p* < 0.05, Figure 4). Similarly, the level of ACH also changed significantly since 7‐day intervention (*p* < 0.05, Figure 4). GABA is the most widely distributed inhibitory neurotransmitter in the central nervous system, which is formed by the removal of the carboxyl group of the excitatory neurotransmitter glutamate in the brain under the action of glutamate decarboxylase (GAD). When mice were irradiated, GABA was significantly increased, while *AS* extract effectively suppressed the increase. The results showed that the inhibitory effect was significant for 3 days of the administration, and with the increase of administration days, the GABA content had an upward trend (Figure 4).

### 3.5 Effects of *Acanthopanax senticosus* extracts on colonic neurotransmitters in irradiated mice

**FIGURE 5:**
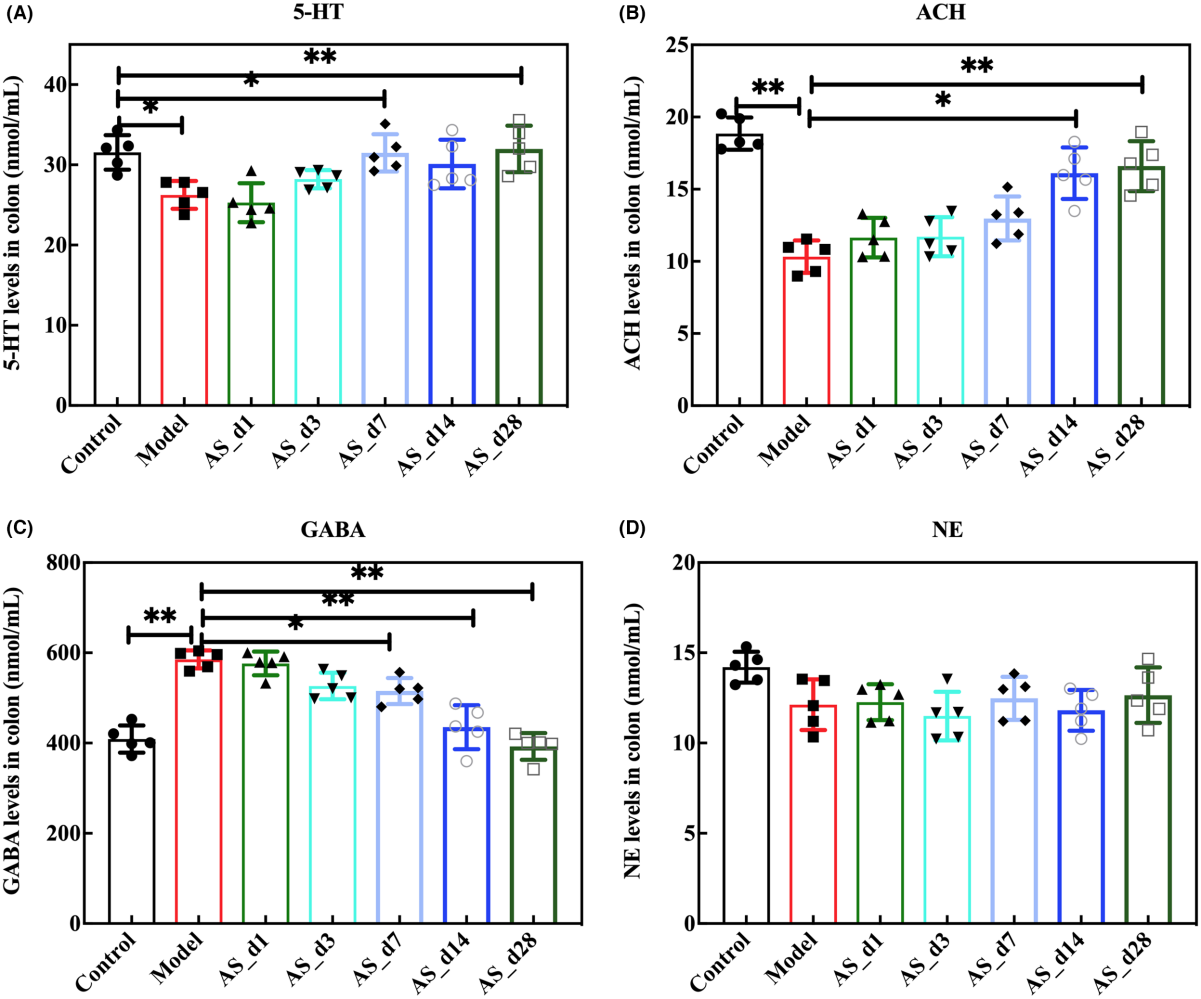
The supplementation of *AS* extract changed the levels of colonic neurotransmitters. (A) The level of 5‐HT; (B) The level of ACH; (C) The level of GABA; (D) The level of NE; Values are expressed as the mean ± SEM (*n* = 5). Statistical analyses were conducted using the one‐way ANOVA followed by Tukey's pos hoc test, *p < 0.05, **p < 0.01).

Intestinal motor function is mainly regulated by the local enteric nervous system (ENS), and the lack or dysfunction of ENS can lead to intestinal dysfunction. In recent years, systematic studies on ENS have proved that there were many neurotransmitters in the gastrointestinal intermuscular plexus and submucosal plexus, which regulated the secretion and motility of the gastrointestinal tract.^25^ It can be seen from Figure 5 that radiation caused changes in neurotransmitters in the colon of mice. However, *AS* intervention changed the content of neurotransmitters in the colon. ACH significantly changed at 14 days of intervention, and GABA and 5‐HT significantly changed at 7 days of intervention (*p* < 0.05, Figure 5). There was no obvious change in NE. It suggested that *AS* may affect the changes of neurotransmitters by changing the intestinal endocrine and movement. Given that the gut microbiota was also closely related to neurotransmitters, we next analyzed the changes in the gut microbiota.

### 3.6 *Acanthopanax senticosus* extract affected the gut microbiota of irradiated mice

**FIGURE 6:**
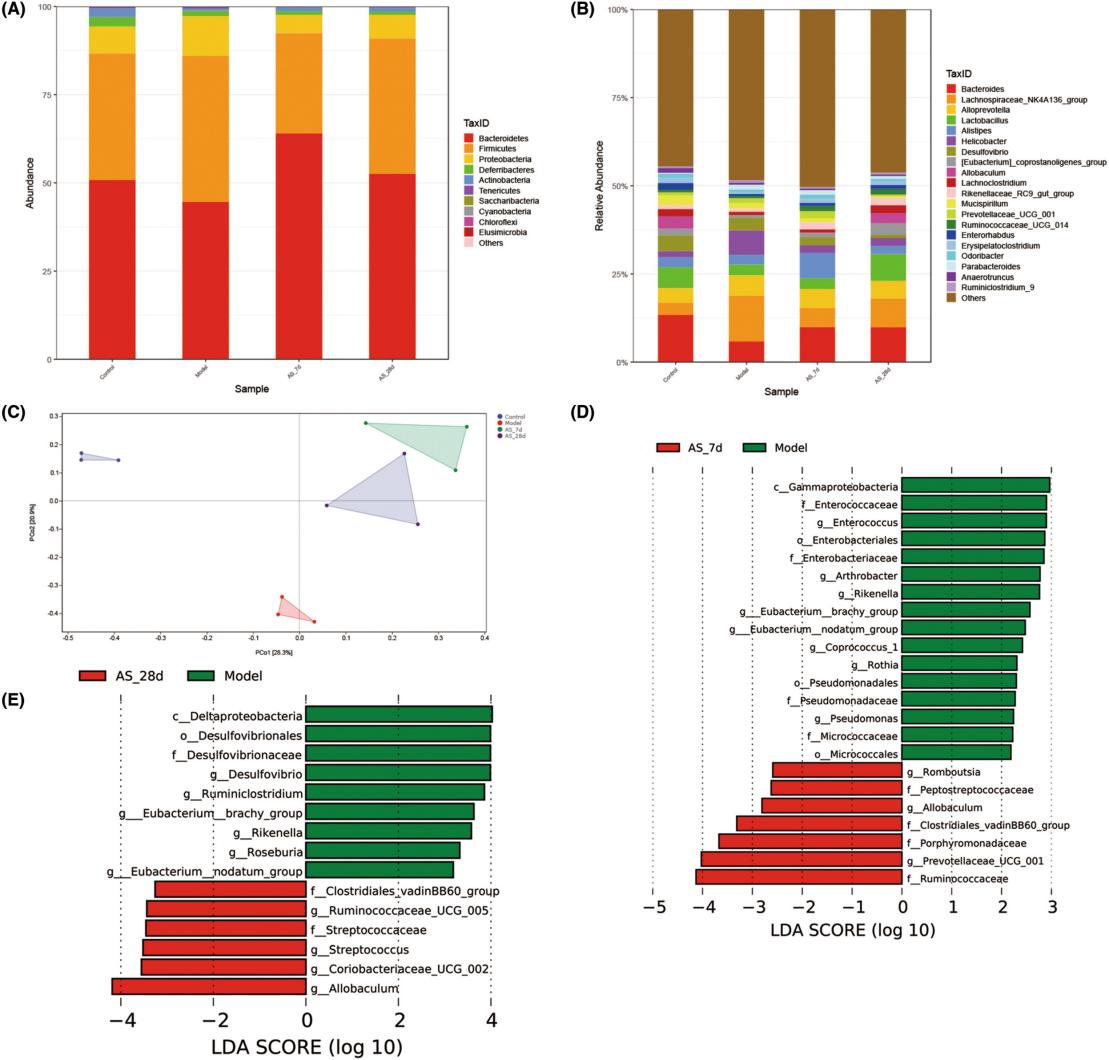
*AS* extract reshaped the gut microbiota composition. (A) The relative abundance of bacteria at the phylum level. (B) The relative abundance of bacteria at the genus level. (C) Principal coordinates analysis plot of Bray‐Curtis. (D) Linear discriminant analysis (LDA) effect size shows the most differentially significant abundant taxa enriched in microbiota from the Model vs. AS_7d. (E) Linear discriminant analysis (LDA) effect size between Model and AS_28d (LDA > 3) (*n* = 3).

Given the above results, there were significant changes in neurotransmitters related to learning and memory, and we found that such changes appeared within 7 days of *AS* intervention. Therefore, we selected the feces of mice treated with *AS* for 7 days and 28 days for intestinal microbiota analysis (Figure 6 and Figure S3). At the phylum level, the domain bacteria were *Bacteroidetes*, *Firmicutes*, *Proteobacteria*, and *Deferribacteres* (Figure 6A). At the genus level, the top 6 relative abundances are *Bacteroides*, *Lachnospiraceae_NK4A136_group*, *Alloprevotella*, *Lactobacillus*, *Alistipes*, and *Helicobacter* (Figure 6B). Radiation reduced the relative abundance of *Bacteroides*, *Lactobacillus*. The relative abundance of *Helicobacter* began to decrease after 7 days of *AS* extract supplementation, and the beneficial bacteria *Lactobacillus* began to increase after 28 days. Meanwhile, PCoA results showed that the four groups of samples were separated from each other, which also proved that the addition of *AS* extract changed the composition of intestinal microbiota in mice (Figure 6C). Lefse analysis was performed to find the key bactreia after radiation and *AS* extract intervention. The results revealed that the marker bacteria supplemented with *AS* extract 7 days were *Romboutsia*, *Allobaculum*, *Prevotellaceae_UCG_001* at genus level and *Peptostreptococcacea*e, *Clostridiales_vadinBB60_group*, *Porphyromonadaceae*, *Ruminococcaceae* at the family level (Figure 6D). Moreover, the marker bacteria of AS_28d group were *Ruminococcaceae_UCG_005*, *Streptococcus*, *Coriobacteriaceae_UCG_002*, *Allobaculum* at the genus level and *Clostridiales_vadinBB60_group*, *Streptococcaceae* at the family level (Figure 6E).

### 3.7 Association of learning and memory‐related neurotransmitters with intestinal microbiota in mice

**FIGURE 7:**
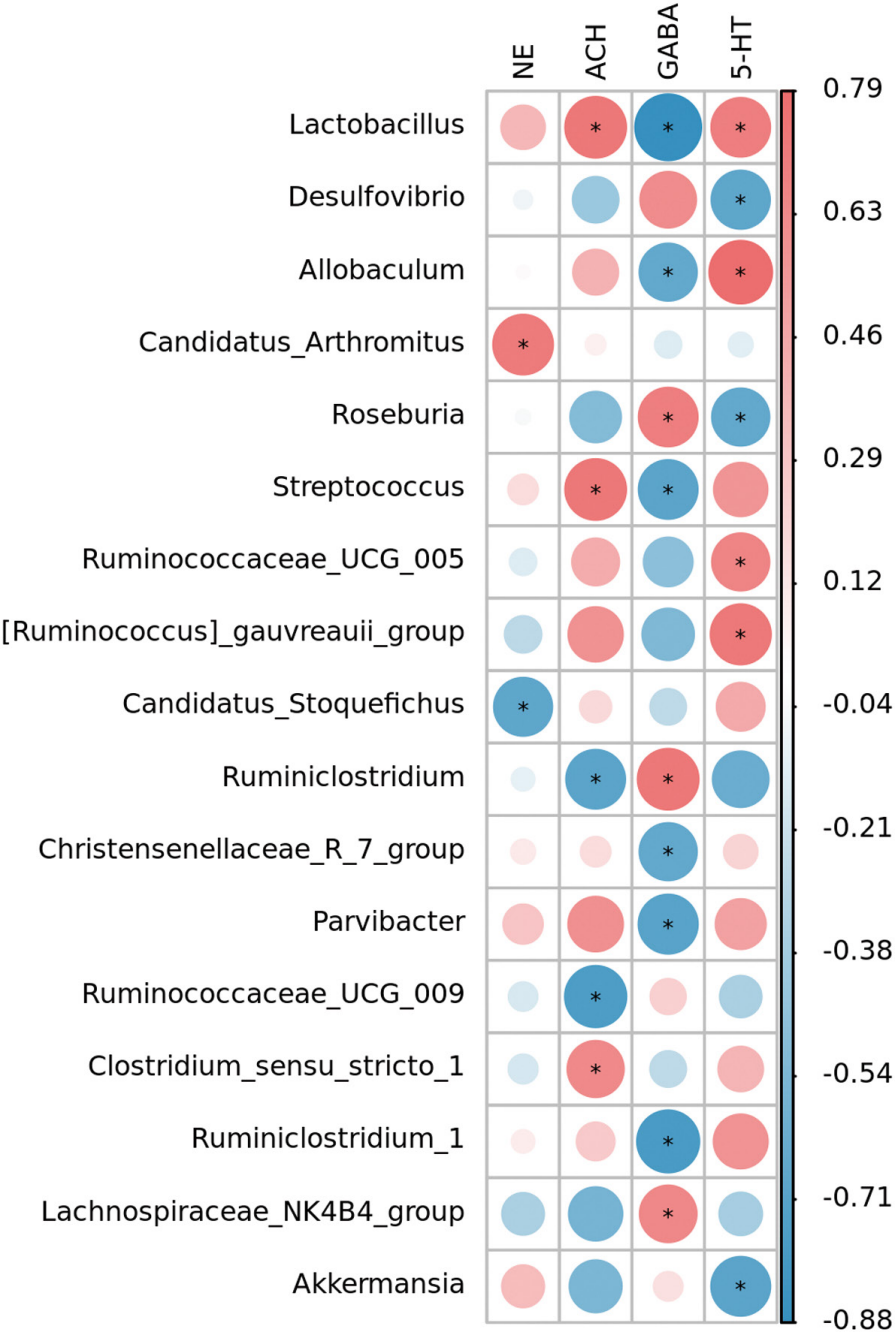
Correlation analysis among the gut microbiota and neurotransmitters. Correlation between gut microbiota and neurotransmitters after 28 days of *AS* extract supplementation. Spearman's correlation analysis was performed by data of the Model group and AS_28d group (*n* = 5). The size of the circle and the color shade represent the value of the correlation coefficient *R*. The larger the circle and the larger the *R*. Red represents positive correlation, blue represents negative correlation, and * represents *p* < 0.05.

Studies have shown that the synthesis of some neurotransmitters is closely related to gut microbiota. We analyzed the correlation between intestinal flora and neurotransmitters after 28 days of intervention with *AS* extract. The results are shown in Figure 7. 5‐HT showed a significant positive correlation with the relative abundance of *Lactobacillus*, *Allobaculum*, *Lachnospiraceae_UCG_005*, *[Ruminococcus]_gauvreauii_group* (*p* < 0.05). Significant negative correlations were found with the relative abundance of *Desulfovibrio*, *Roseburia*, *Akkermansia* (*p* < 0.05). The genera with significant positive correlation with ACH were *Lactobacillus*, *Streptococcus*, *Clostridium_sensu_stricto_1*, *Ruminiclostridium*, *Ruminococcaceae_UCG_009* (*p* < 0.05). The relative abundance of *Candidatus_ Arthromitus* was significantly positively correlated with NE and *Candidatus_Stoquefichus* was significantly negatively correlated with NE (*p* < 0.05). The relative abundance of *Roseburia*, *Ruminiclostridium*, and *Lachnospiraceae_NK4B4_group* was significantly positively correlated with GABA (*p* < 0.05).

### 3.8 *Acanthopanax senticosus* supplementation decreased Inflammatory cytokines and enhanced tight junction

**FIGURE 8:**
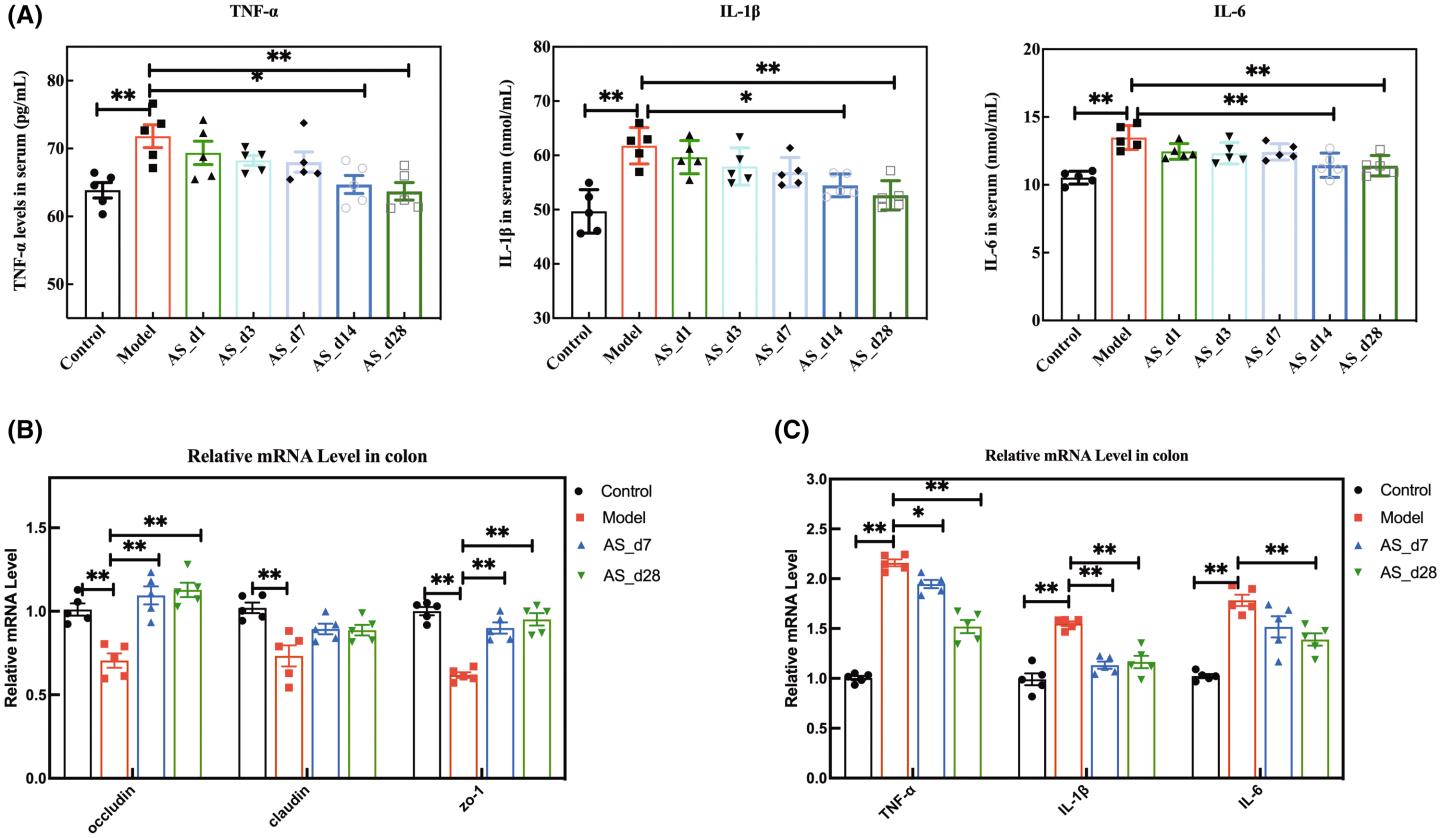
*AS* extract prevented inflammation levels and colonic mucosa barrier impairment in irradiated mice. (A) The level of Inflammatory cytokines in serum. (B) The mRNA expression of inflammatory cytokines in the colon. (C) The mRNA expression of tight junction proteins in the colon of mice. (Values are expressed as the mean ± SEM (n = 5). Statistical analyses were conducted using the one‐way ANOVA followed by Tukey's pos hoc test, *p < 0.05, **p < 0.01).

We then detected the mRNA expression of colonic inflammatory factors and tight junction proteins. We found that intervention with *Acanthopanax senticosus* extract reduced the expression levels of TNF‐α, IL‐6, and IL‐1β in the colon, while also increasing the expression levels of tight junction protein (Figure 8B). It is also one of the reasons for the changes in the intestinal microbiota. In addition, we detected the levels of inflammatory factors in the serum. The intervention of the *AS* extract also reduced radiation‐induced inflammation (Figure 8A). The intake of *Acanthopanax senticosus* extract may affect radiation‐induced systemic inflammatory response and even neuroinflammation.

### 3.9 *Acanthopanax senticosus* supplementation increased the relative protein expression of BDNF

**FIGURE 9:**
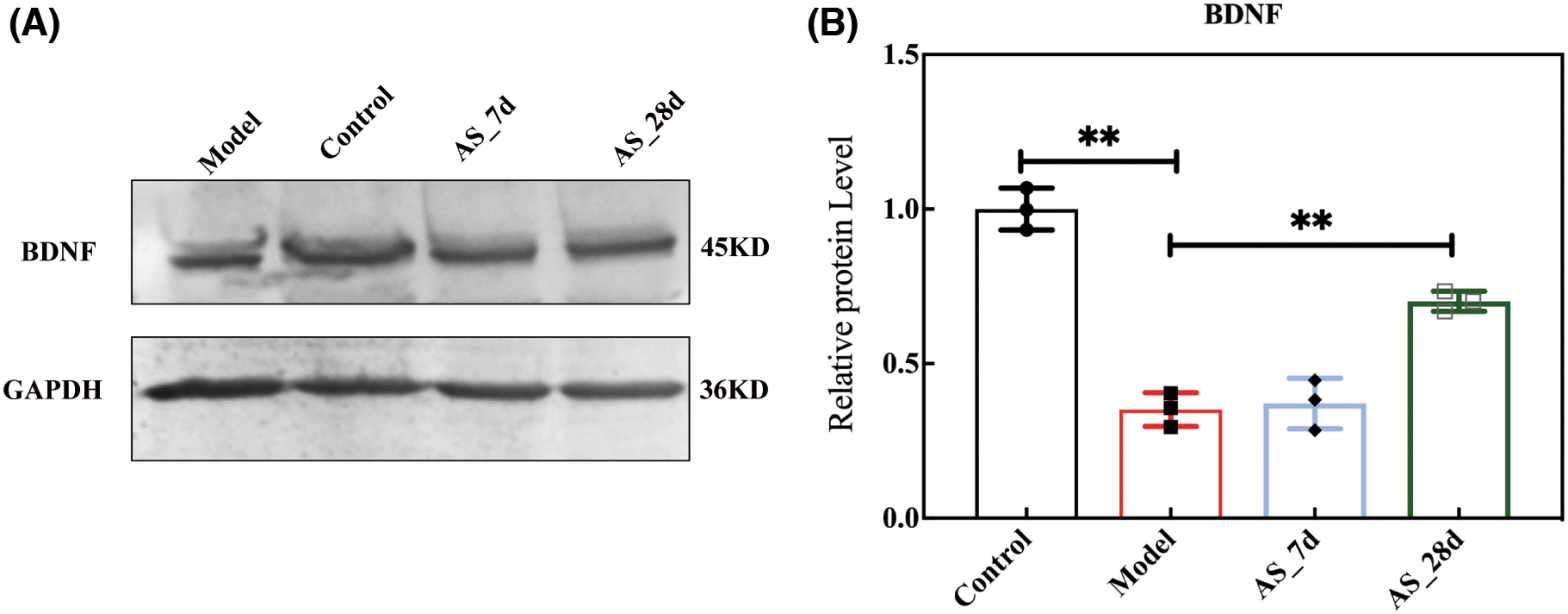
*AS* supplementation increased the protein level of BDNF. (A) The image of protein bands. (B) The relative protein expression of BDNF. Values are expressed as the mean ± SEM (*n* = 3). Statistical analyses were conducted using the one‐way ANOVA followed by Tukey's pos hoc test, **p < 0.01).

BDNF (Brain‐derived neurotrophic factor) has a wide range of functions in the brain, participating in physiological processes such as neuroplasticity, neuronal survival, synapse formation, and neurotransmitter regulation.^26^, ^27^ In the above results, we observed the modulation of neurotransmitters and neuroinflammation by gut microbiota. Now the expression of BDNF at the protein level in the hippocampus was examined. The results showed that radiation exposure decreased the expression of BDNF and *AS* extract supplementation for 7 days could not significantly increase the expression of BDNF. However, *AS* extract supplementation for 28 days could significantly increase the expression of BDNF (Figure 9). *AS* extract supplementation may improve learning and memory ability by increasing the expression of BDNF.

### 3.10 *Acanthopanax senticosus* supplementation inhibited the neuroinflammation

**FIGURE 10:**
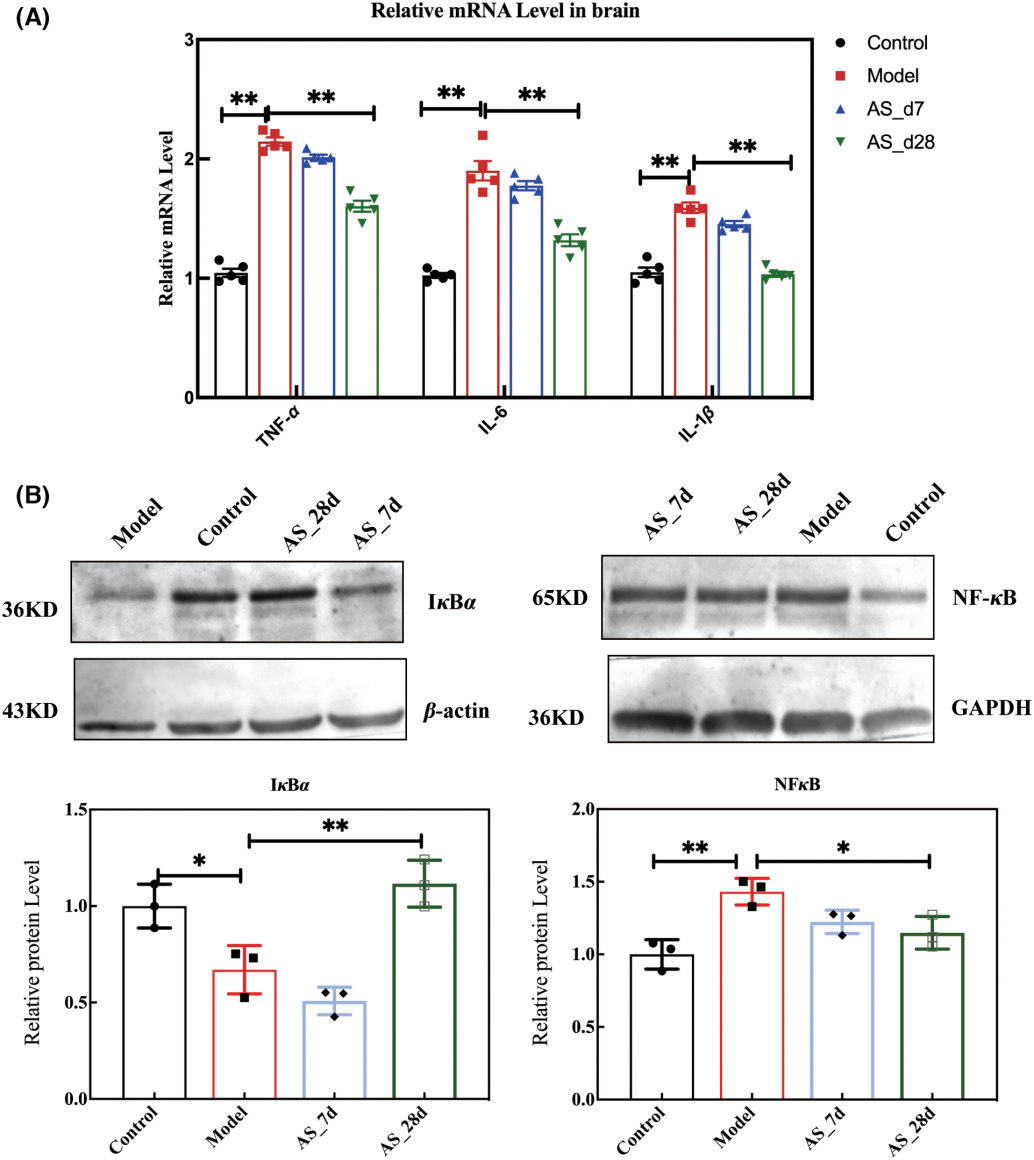
*AS* supplementation inhibited neuroinflammation. (A) The relative expression of inflammatory cytokines in the brain. (B) The relative protein expression of NF‐κB and IκBα. Values are expressed as the mean ± SEM (*n* = 3). Statistical analyses were conducted using the one‐way ANOVA followed by Tukey's pos hoc test, **p < 0.01).

Radiation‐induced systemic inflammation leads to neuroinflammation, and we examined the expression of mRNA levels of inflammatory factors in the hippocampus. The results are shown in Figure 10A. The intervention of Acanthopanax extract for 28 days significantly reduced the expression of TNF‐α, IL‐6, and IL‐1β in the hippocampus (*p* < 0.01). We also investigated the expression of NF‐κB protein. Through western blot experiments, we found that radiation significantly increased the relative protein expression of NF‐κB and significantly decreased the relative protein expression of IκBα compared with the control group (*p* < 0.01). The intervention of AS extract for 28 days significantly reduced the expression of NF‐κB (*p* < 0.05) and elevated the expression of IκBα (*p* < 0.01). AS extract inhibited the activation of NF‐κB.

## 4 DISCUSSION

The brain is highly sensitive to radiation, which usually causes a decline in learning and memory.^28^, ^29^ Our previous study also confirmed this conclusion.^30^ *AS* is a kind of wild herb with the homology of medicine and food. This study mainly explored the protective effect of *As* extract on learning and memory impairment in irradiated mice under different days of dietary intervention. Many natural products are known for be radiation resistant, such as polysaccharides, saponins, and flavonoids.^31^, ^32^ The type, content and functionality of the active ingredients contained in different parts of AS differ. The roots and stems of AS mainly include syringin, eleutheroside D,eleutheroside E, and isofraxidin. The leaves and fruit of AS mainly contain flavonoids, saponins and organic acids. The active ingredients are closely related to anti‐stress, anti‐inflammatory and neuroprotective effects.^33^ In this study, *AS* extract improved the decline in learning and memory ability of mice caused by radiation and also changed the level of neurotransmitters (Figures 2, 4 and 5). Some other studies have also reported changes of neurotransmitters in the brain after taking Chinese herbal medicine.^34^, ^35^ Neurotransmitters are chemicals that travel across synapses between neurons, transmitting information to control movement, mood, memory, and other behaviors. These chemical messengers have excitatory or inhibitory effects on neurons. In this experiment, we found that different dosing cycles affected neurotransmitter levels. Neurotransmitters started to change after 7 days of administration.

It is known that radiation also causes changes in gut microbiota, and many current studies have focused on the mechanisms underlying the brain‐gut axis. The AS extract contains polysaccharides, flavonoids, saponins, amino acids, and so on. The active components can interact with intestinal microbiota and exert physiological activities. On the one hand, the gut microbiota can metabolize some active components into new metabolites to play a role. For example, flavonoids can be converted into phenolic acids by the action of intestinal microbiota through enzymatic, hydrolytic, and dehydroxylation reactions to improve bioavailability. Glycosides are not easily absorbed in the intestinal tract, but can be metabolized by intestinal microbiota to deglycosylate glycosides, thus improving bioavailability. *Bifidobacterium*, *Lactobacillus*, *Lachnospira*, and *Enterococcus* efficiently participated in the deglycosylation hydrolysis reaction of the glycosides, while *Bacillus* and *Clostridium* can further degrade the sapogenins. Alkaloids are easily converted into metabolites such as monoester‐type alkaloids by anaerobic bacteria in the intestinal tract. In the colon, intestinal microbes can metabolize food that is not absorbed by the small intestine and produce a range of metabolites, such as short‐chain fatty acids, vitamins, and secondary bile salts.^36^ On the other hand, active compounds can regulate the composition of the gut microbiota. For example, polysaccharide components have a prebiotic effect, which can promote the growth of beneficial bacteria and the metabolites can inhibit the reproduction of harmful bacteria. Moreover, plant extracts restored the diversity of gut microbiota and enhanced the growth of beneficial bacteria such as *Lactobacillus* and *Bifidobacterium*. In our study, AS extract intervention increased the relative abundance of *Lactobacillus*. Thirdly, plant extracts protect the intestinal barrier function by regulating flora and preventing bacterial translocation. Together, these active ingredients can play a role by regulating the gut microbiota in both direct and indirect ways. In the present study, the supplementation of AS extract enhanced the intestinal barrier function (Figure 8). The marker bacteria in the *AS* extract group played an important role in learning and memory ability. Probiotic mixtures of *Lactobacillus* and *Bifidobacterium* strains significantly improved spatial memory deficits in diabetic animals,^37^ and *Streptococcus* supplementation restored learning memory deficits.^38^ Moreover, *Lactobacillus* and *Streptococcus* are involved in the synthesis of 5‐HT and ACH. *Allobaculum* was reduced in depressed patients and probiotic supplementation improved *Ruminococcaceae* restoring cognitive deficits, after 28 days of *Streptococcus* supplementation.^39^ We observed an increase in the above genus, thus playing a role in learning memory. In addition, we speculated that the neurotransmitter changes induced by the intervention of *AS* extract may be closely related to the altered gut microbiota. The gut flora digests the host's dietary components while providing the host with energy and nutrients. The gut microbiota also produces some neuroactive metabolites, which can cause changes in neurotransmitters or their precursors.^40^, ^41^ Ruminococcus is involved in the synthesis of tryptamine, which induces 5‐HT secretion.^42^, ^43^ Clostridiales play an important role in stimulating the biosynthesis and release of serotonin (5‐HT) from intestinal enterochromaffin cells and modulating GI motility.^44^ In this study, the abundance of Ruminococcus and Clostridiales changed significantly after 7 days of intervention by *AS* extract, accompanied by changes in 5‐HT level (Figures 4 and 5). In addition, it has been reported that Lactobacillus and Streptococcus are involved in the synthesis of 5‐HT and ACH.^45^, ^46^, ^47^ After the intervention of *AS* extract for 28 days, the abundance of Streptococcus increased, as well as increased the level of 5‐HT and ACH. But the level of GABA decreased. Since neurotransmitters cannot directly penetrate the blood–brain barrier,^48^ we speculated that the changes in neurotransmitters caused by *AS* were due to bacterial synthesis of neurotransmitters on the one hand, and neurotransmitter precursor substances produced by microbial metabolites to enter the blood–brain barrier on the other hand. Some studies have confirmed that metabolites or molecules of gut microbiota can travel up the vagus nerve to the brain, but it is unclear whether neurotransmitters produced in the gut can be transported to the brain through the vagus nerve.^49^ In a Chinese patient group presenting with stroke or a transient ischemic attack, the fecal abundance of *Prevotella* was severely depleted.^50^ In addition, the abundance of *Prevotellaceae* decreased in the PD patient.^51^7‐day intervention with *AS* extracts induced changes in *Prevotellaceae* in our study. In conclusion, *Acanthopanax senticosus* extracts caused changes in the composition of gut microbiota, while also changing neurotransmitters and there was a correlation between the two. Therefore, we hypothesized that *AS* extract prevents radiation‐induced brain injury through neurotransmitter ‐ microbiota communication. In addition, the destruction of intestinal tight junctions and the increase of inflammatory factors can lead to changes in intestinal permeability and thus changes in the gut microbiota. In this study, the *AS* extract also had a good protective effect on the intestinal tract. At the same time, it can also reduce the inflammatory factors of serum. Microglia is the resident innate immune cells in the brain, which respond to various stimuli that trigger the release of proinflammatory factors that could potentially damage the brain.^52^ Furthermore, microglia express a variety of neurotransmitters, neuropeptides, and immune receptors and have the capacity to rapidly respond to physiological changes in the brain.^53^ We have observed elevated inflammatory factors in serum, and studies have shown that systemic inflammation often has a dramatic impact on cognition and behavior. Free radicals and cytokines, such as interleukins (ILs) and tumor necrosis factor‐alpha (TNF‐α), are released by microglia in response to neurotransmitter stimulation and have profound effects on neurons. BDNF is considered as a possible biomarker to monitor the onset, progression and treatment of brain lesions.^54^ The role of BDNF in neuroinflammation is strongly related to its ability to induce—and be induced by—NF‐κB.^55^ In the resting state, NF‐κB is present in the cytoplasm in an inactive form, bound to its repressor protein. Upon upstream stimulatory signals, IκB is phosphorylated and degraded, activating NF‐κB, which initiates transcription and release of inflammatory factors. The released inflammatory factors, TNF‐α, IL‐1β and IL‐6, in turn, act to activate NF‐κB, thus constituting positive feedback that amplifies the inflammatory response cascade. The intervention of Acanthopanax extract inhibited the activation of NF‐κB and suppressed neuroinflammation. Given that *AS* extract reduced the level of inflammation and alleviated brain damage in irradiated mice, our future studies will focus on its interaction in regulating neurotransmitter and microglia activation and neuroinflammation.

## 5 CONCLUSION

In summary, the current research suggested *AS* extract improved cognitive function in a novel way and found a link between gut microbiota and cognitive function via neurotransmitters. Furthermore, *AS* extract reduced the level of inflammation and our future studies can focus on its interaction in regulating neurotransmitter and microglia activation. It may be the potential mechanism that *Acanthopanax senticosus* extract ameliorates radiation‐induced brain injury.

## CONFLICT OF INTEREST STATEMENT

The authors declare that they have no competing interests.

## Supporting information


Figure S1

Figure S2

Figure S3

## Data Availability

The data that support the findings of this study are available from the corresponding author upon reasonable request. The original blots images can be available in the Figures S1 and S2.

## References

[1] Balentova S , Adamkov M . Pathological changes in the central nervous system following exposure to ionizing radiation. Physiol Res. 2020;69(3):389‐404. 32469226 10.33549/physiolres.934309 PMC8648310

[2] Dietrich J , Baryawno N , Nayyar N , et al. Bone marrow drives central nervous system regeneration after radiation injury. J Clin Investig. 2018;128(6):2651. 10.1172/JCI121592 PMC5983336 29856368

[3] Helson L . Radiation‐induced demyelination and remyelination in the central nervous system: a literature review. Anticancer Res. 2018;38(9):4999‐5002. 30194143 10.21873/anticanres.12818

[4] Wang Q‐Q , Yin G , Huang J‐R , et al. Ionizing radiation‐induced brain cell aging and the potential underlying molecular mechanisms. Cell. 2021;10(12):3570. 10.3390/cells10123570 PMC8700624 34944078

[5] Huang S , Chen F , Cheng H , Huang G . Modification and application of polysaccharide from traditional Chinese medicine such as Dendrobium officinale. Int J Biol Macromol. 2020;157:385‐393. 32339575 10.1016/j.ijbiomac.2020.04.141

[6] Aygun B , Alaylar B , Turhan K , et al. Investigation of neutron and gamma radiation protective characteristics of synthesized quinoline derivatives. Int J Radiat Biol. 2020;96(11):1423‐1434. 32813583 10.1080/09553002.2020.1811421

[7] Liu X , Chu W , Shang S , et al. Preliminary study on the anti‐apoptotic mechanism of Astragaloside IV on radiation‐induced brain cells. Int J Immunopathol Pharmacol. 2020;34:205873842095459. 10.1177/2058738420954594 PMC7485151 32902354

[8] Liu S‐m , Li X‐z , Huo Y , Lu F . Protective effect of extract of Acanthopanax senticosus harms on dopaminergic neurons in Parkinson's disease mice. Phytomedicine. 2012;19(7):631‐638. 22402244 10.1016/j.phymed.2012.02.006

[9] Xu Y , Han C , Xu S , Yu X , Jiang G , Nan C . Effects of Acanthopanax senticosus on learning and memory in a mouse model of Alzheimer's disease and protection against free radical injury to brain tissue. Neural Regen Res. 2008;3(2):192‐195.

[10] Lin B , Chen R , Wang Q , Li Z , Yang S , Feng Y . Transcriptomic and metabolomic profiling reveals the protective effect of *Acanthopanax senticosus* (Rupr. & Maxim.) Harms combined with *Gastrodia elata* Blume on cerebral ischemia–reperfusion injury. Front Pharmacol. 2021;12:619076. 33935709 10.3389/fphar.2021.619076 PMC8085551

[11] Clarke G , Grenham S , Scully P , et al. The microbiome‐gut‐brain axis during early life regulates the hippocampal serotonergic system in a sex‐dependent manner. Mol Psychiatry. 2013;18(6):666‐673. 22688187 10.1038/mp.2012.77

[12] Lai K‐P , Chung Y‐T , Li R , Wan H‐T , Wong CK‐C . Bisphenol a alters gut microbiome: comparative metagenomics analysis. Environ Pollut. 2016;218:923‐930. 27554980 10.1016/j.envpol.2016.08.039

[13] Adami R , Bottai D . Movement impairment: focus on the brain. J Neurosci Res. 2016;94(4):310‐317. 26762181 10.1002/jnr.23711

[14] Wilck N , Matus MG , Kearney SM , et al. Salt‐responsive gut commensal modulates TH17 axis and disease. Nature. 2017;551(7682):585‐589. 29143823 10.1038/nature24628 PMC6070150

[15] David LA , Maurice CF , Carmody RN , et al. Diet rapidly and reproducibly alters the human gut microbiome. Nature. 2014;505(7484):559‐563. 24336217 10.1038/nature12820 PMC3957428

[16] Dominguez‐Bello MG , Costello EK , Contreras M , et al. Delivery mode shapes the acquisition and structure of the initial microbiota across multiple body habitats in newborns. Proc Natl Acad Sci. 2010;107(26):11971‐11975. 20566857 10.1073/pnas.1002601107 PMC2900693

[17] McEwen BS , Nasca C , Gray JD . Stress effects on neuronal structure: hippocampus, amygdala, and prefrontal cortex. Neuropsychopharmacology. 2016;41(1):3‐23. 26076834 10.1038/npp.2015.171 PMC4677120

[18] Mayer EA , Tillisch K , Gupta A . Gut/brain axis and the microbiota. J Clin Invest. 2015;125(3):926‐938. 25689247 10.1172/JCI76304 PMC4362231

[19] Song W , Shi J , Baranenko D , Jing J , Lu W . Radioprotective effects of active compounds of Acanthopanax senticosus from the lesser Khingan Mountain range in China. RSC Adv. 2016;6(1):65‐72.

[20] Wang Q , Garrity GM , Tiedje JM , Cole JR . Naive Bayesian classifier for rapid assignment of rRNA sequences into the new bacterial taxonomy. Appl Environ Microbiol. 2007;73(16):5261‐5267. 17586664 10.1128/AEM.00062-07 PMC1950982

[21] Li X , Fan P , Zang M , Xing J . Rapid determination of oligopeptides and amino acids in soybean protein hydrolysates using high‐resolution mass spectrometry. Phytochem Anal. 2015;26(1):15‐22. 25043352 10.1002/pca.2531

[22] Clifford MN , Johnston KL , Knight S , Kuhnert N . Hierarchical scheme for LC‐MSn identification of chlorogenic acids. J Agric Food Chem. 2003;51(10):2900‐2911. 12720369 10.1021/jf026187q

[23] Slacanin I , Marston A , Hostettmann K , Guedon D , Abbe P . The isolation of Eleutherococcus senticosus constituents by centrifugal partition chromatography and their quantitative determination by high performance liquid chromatography. Phytochem Anal. 1991;2(3):137‐142.

[24] Wang Y‐H , Meng Y , Zhai C , et al. The chemical characterization of *Eleutherococcus senticosus* and ci‐wu‐jia tea using UHPLC‐UV‐QTOF/MS. Int J Mol Sci. 2019;20(3):475. 30678313 10.3390/ijms20030475 PMC6387334

[25] Fournel A , Drougard A , Duparc T , et al. Apelin targets gut contraction to control glucose metabolism via the brain. Gut. 2017;66(2):258‐269. 26565000 10.1136/gutjnl-2015-310230 PMC5284480

[26] Edelmann E , Lessmann V , Brigadski T . Pre‐ and postsynaptic twists in BDNF secretion and action in synaptic plasticity. Neuropharmacology. 2014;76:610‐627. 23791959 10.1016/j.neuropharm.2013.05.043

[27] Panja D , Bramham CR . BDNF mechanisms in late LTP formation: a synthesis and breakdown. Neuropharmacology. 2014;76:664‐676. 23831365 10.1016/j.neuropharm.2013.06.024

[28] Monje ML , Mizumatsu S , Fike JR , Palmer TD . Irradiation induces neural precursor‐cell dysfunction. Nat Med. 2002;8(9):955‐962. 12161748 10.1038/nm749

[29] Greene‐Schloesser D , Robbins ME , Peiffer AM , Shaw EG , Wheeler KT , Chan MD . Radiation‐induced brain injury: a review. Front Oncol. 2012;2:73. 22833841 10.3389/fonc.2012.00073 PMC3400082

[30] Song C , Gao X , Song W , et al. Simulated spatial radiation impacts learning and memory ability with alterations of neuromorphology and gut microbiota in mice. RSC Adv. 2020;10(27):16196‐16208. 35493686 10.1039/d0ra01017k PMC9052872

[31] Yi J , Zhu J , Zhao C , et al. Potential of natural products as radioprotectors and radiosensitizers: opportunities and challenges. Food Funct. 2021;12(12):5204‐5218. 34018510 10.1039/d1fo00525a

[32] Wang Q , Xie C , Xi S , et al. Radioprotective effect of flavonoids on ionizing radiation‐induced brain damage. Molecules. 2020;25(23):5719. 33287417 10.3390/molecules25235719 PMC7730479

[33] R‐h C , W‐d D , Wang Q , et al. Effects of *Acanthopanax senticosus* (Rupr. & Maxim.) Harms on cerebral ischemia–reperfusion injury revealed by metabolomics and transcriptomics. J Ethnopharmacol. 2021;264:113212. 32768643 10.1016/j.jep.2020.113212

[34] Hu Y , Wang YN , Zhang GQ , Dong XZ , Liu WW , Liu P . Gan‐dan‐Liang‐Yi‐Tang alleviates p‐chlorophenylalanine‐induced insomnia through modification of the serotonergic and immune system. Exp Ther Med. 2016;12(5):3087‐3092. 27882122 10.3892/etm.2016.3737 PMC5103753

[35] Zhou Q‐H , Zhou X‐L , Xu M‐B , et al. Suanzaoren formulae for insomnia: updated clinical evidence and possible mechanisms. Front Pharmacol. 2018;9:. 10.3389/fphar.2018.00076 PMC5811769 29479317

[36] Nicholson JK , Holmes E , Kinross J , et al. Host‐gut microbiota metabolic interactions. Science. 2012;336(6086):1262‐1267. 22674330 10.1126/science.1223813

[37] Davari S , Talaei SA , Alaei H , Salami M . Probiotics treatment improves diabetes‐induced impairment of synaptic activity and cognitive function: behavioral and electrophysiological proofs for microbiome‐gut‐brain axis. Neuroscience. 2013;240:287‐296. 23500100 10.1016/j.neuroscience.2013.02.055

[38] Beilharz JE , Kaakoush NO , Maniam J , Morris MJ . Cafeteria diet and probiotic therapy: cross talk among memory, neuroplasticity, serotonin receptors and gut microbiota in the rat. Mol Psychiatry. 2018;23(2):351‐361. 28289278 10.1038/mp.2017.38

[39] Higarza SG , Arboleya S , Arias JL , Gueimonde M , Arias N . Akkermansia muciniphila and environmental enrichment reverse cognitive impairment associated with high‐fat high‐cholesterol consumption in rats. Gut Microbes. 2021;13(1):1‐20. 10.1080/19490976.2021.1880240 PMC7946069 33678110

[40] Frost G , Sleeth ML , Sahuri‐Arisoylu M , et al. The short‐chain fatty acid acetate reduces appetite via a central homeostatic mechanism. Nature Communications. 2014;5(1):3611. 10.1038/ncomms4611 PMC4015327 24781306

[41] Gao K , Pi Y , Mu C‐L , Farzi A , Liu Z , Zhu W‐Y . Increasing carbohydrate availability in the hindgut promotes hypothalamic neurotransmitter synthesis: aromatic amino acids linking the microbiota‐brain axis. J Neurochem. 2019;149(5):641‐659. 31006109 10.1111/jnc.14709

[42] Williams BB , Van Benschoten AH , Cimermancic P , et al. Discovery and characterization of gut microbiota decarboxylases that can produce the neurotransmitter tryptamine. Cell Host Microbe. 2014;16(4):495‐503. 25263219 10.1016/j.chom.2014.09.001 PMC4260654

[43] Takaki M , Mawe GM , Barasch JM , Gershon MD , Gershon MD . Physiological responses of Guinea‐pig myenteric neurons secondary to the release of endogenous serotonin by tryptamine. Neuroscience. 1985;16(1):223‐240. 2940472 10.1016/0306-4522(85)90059-4

[44] Labus JS , Osadchiy V , Hsiao EY , et al. Evidence for an association of gut microbial clostridia with brain functional connectivity and gastrointestinal sensorimotor function in patients with irritable bowel syndrome, based on tripartite network analysis. Microbiome. 2019;7(1):45. 30898151 10.1186/s40168-019-0656-z PMC6429755

[45] Ouml Z , Ul F , et al. The function of lactic acid bacteria on biogenic amines production by food‐borne pathogens in arginine decarboxylase broth. Food Sci Technol Res. 2012;18(6):795‐804.

[46] Yang SY , Lü FX , Lu ZX , et al. Production of γ‐aminobutyric acid by *Streptococcus salivarius* subsp. thermophilus Y2 under submerged fermentation. Amino Acids. 2008;34(3):473‐478. 17514494 10.1007/s00726-007-0544-x

[47] Stanaszek PM , Snell JF , O'Neill JJ . Isolation, extraction, and measurement of acetylcholine from lactobacillus plantarum. Appl Environ Microbiol. 1977;34(2):237‐239. 907345 10.1128/aem.34.2.237-239.1977 PMC242629

[48] Chen Y , Xu J , Chen Y . Regulation of neurotransmitters by the gut microbiota and effects on cognition in neurological disorders. Nutrients. 2021;13(6):2099. 34205336 10.3390/nu13062099 PMC8234057

[49] Lee K‐E , Kim J‐K , Han S‐K , et al. The extracellular vesicle of gut microbial Paenalcaligenes hominis is a risk factor for vagus nerve‐mediated cognitive impairment. Microbiome. 2020;8(1):107. 32669127 10.1186/s40168-020-00881-2 PMC7364628

[50] Yin J , Liao SX , He Y , et al. Dysbiosis of gut microbiota with reduced trimethylamine‐N‐oxide level in patients with large‐artery atherosclerotic stroke or transient ischemic attack. J Am Heart Assoc. 2015;4(11):e002699. 26597155 10.1161/JAHA.115.002699 PMC4845212

[51] Bedarf JR , Hildebrand F , Coelho LP , et al. Functional implications of microbial and viral gut metagenome changes in early stage L‐DOPA‐naïve Parkinson's disease patients. Genome Med. 2017;9(1):39. 28449715 10.1186/s13073-017-0428-y PMC5408370

[52] Zhu C , Herrmann US , Falsig J , et al. A neuroprotective role for microglia in prion diseases. J Exp Med. 2016;213(6):1047‐1059. 27185853 10.1084/jem.20151000 PMC4886355

[53] Lewitus GM , Konefal SC , Greenhalgh AD , Pribiag H , Augereau K , Stellwagen D . Microglial TNF‐α suppresses cocaine‐induced plasticity and behavioral sensitization. Neuron. 2016;90(3):483‐491. 27112496 10.1016/j.neuron.2016.03.030 PMC4860141

[54] Nagahara AH , Tuszynski MH . Potential therapeutic uses of BDNF in neurological and psychiatric disorders. Nat Rev Drug Discov. 2011;10(3):209‐219. 21358740 10.1038/nrd3366

[55] Lima Giacobbo B , Doorduin J , Klein HC , Dierckx RAJO , Bromberg E , de Vries EFJ . Brain‐derived neurotrophic factor in brain disorders: focus on neuroinflammation. Mol Neurobiol. 2019;56(5):3295‐3312. 30117106 10.1007/s12035-018-1283-6 PMC6476855

