# Supplementary figures and images for: Acanthopanax senticosus extract alleviates radiation‐induced learning and memory impairment based on neurotransmitter‐gut microbiota communication
Source: CNS Neurosci Ther. 2023 Mar 27;29(Suppl 1):129–45. doi: 10.1111/cns.14134 (PMC10314102; doi:10.1111/cns.14134)

Figure S1

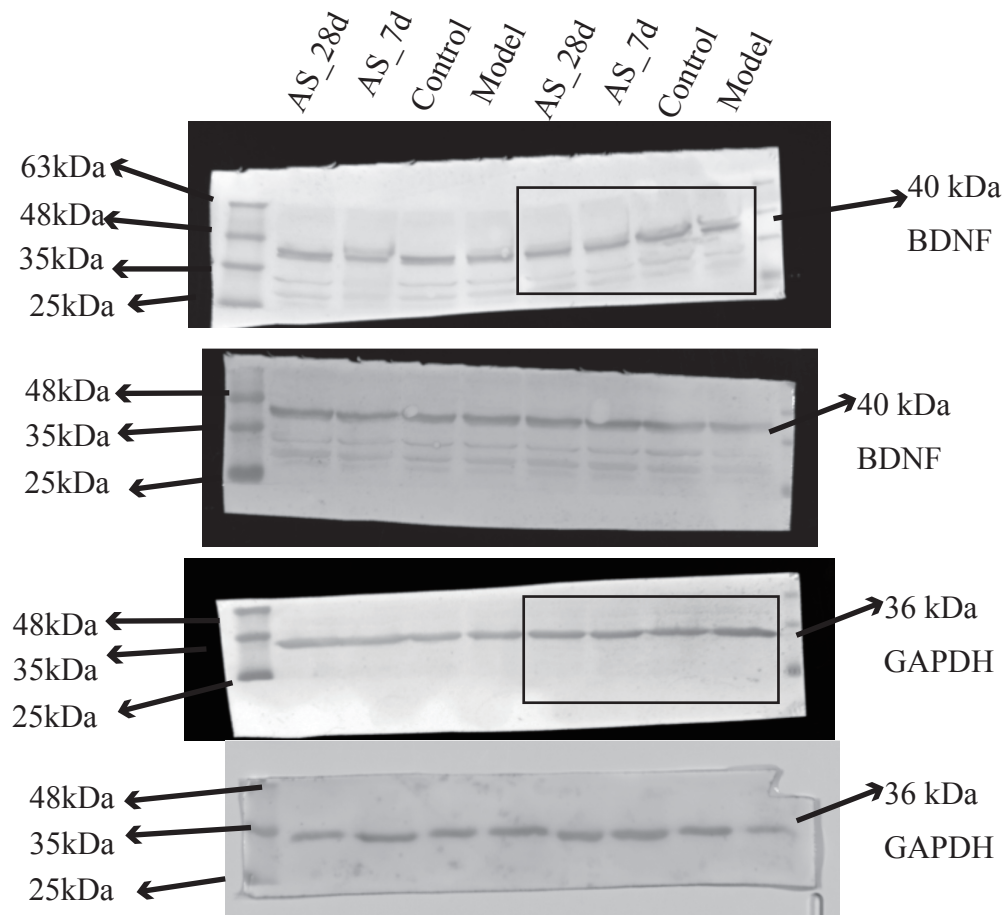

Supplement: Supplementary file 1 — Figure S1 [file CNS-29-129-s001.pdf]

**Figure S2**

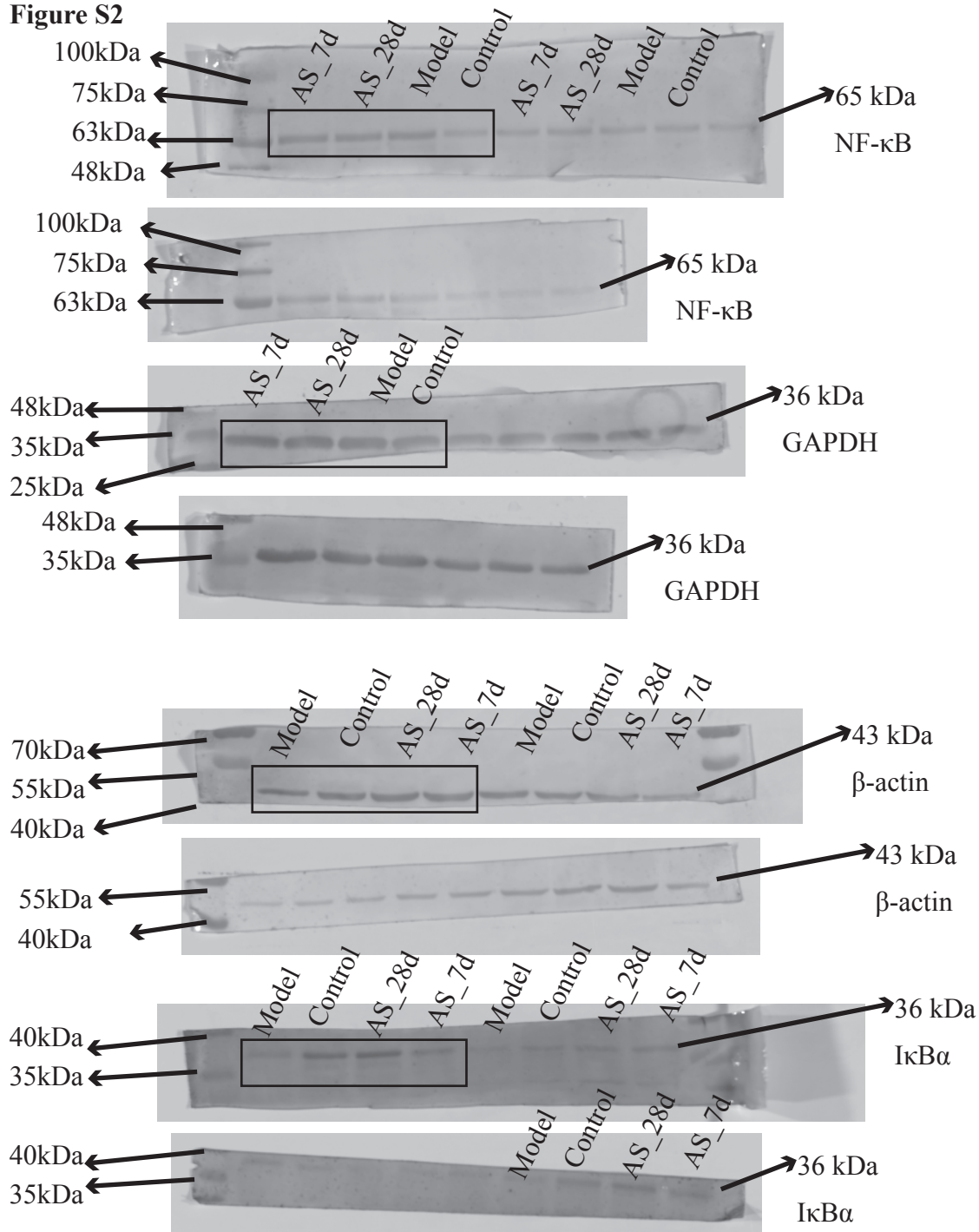

Supplement: Supplementary file 2 — Figure S2 [file CNS-29-129-s002.pdf]
