# Supplementary material for: Acanthopanax senticosus extract alleviates radiation‐induced learning and memory impairment based on neurotransmitter‐gut microbiota communication
Source: CNS Neurosci Ther. 2023 Mar 27;29(Suppl 1):129–45. doi: 10.1111/cns.14134 (PMC10314102; doi:10.1111/cns.14134)
Supplement: Supplementary file 3 — Figure S3 [file CNS-29-129-s003.pdf]

FigureS3

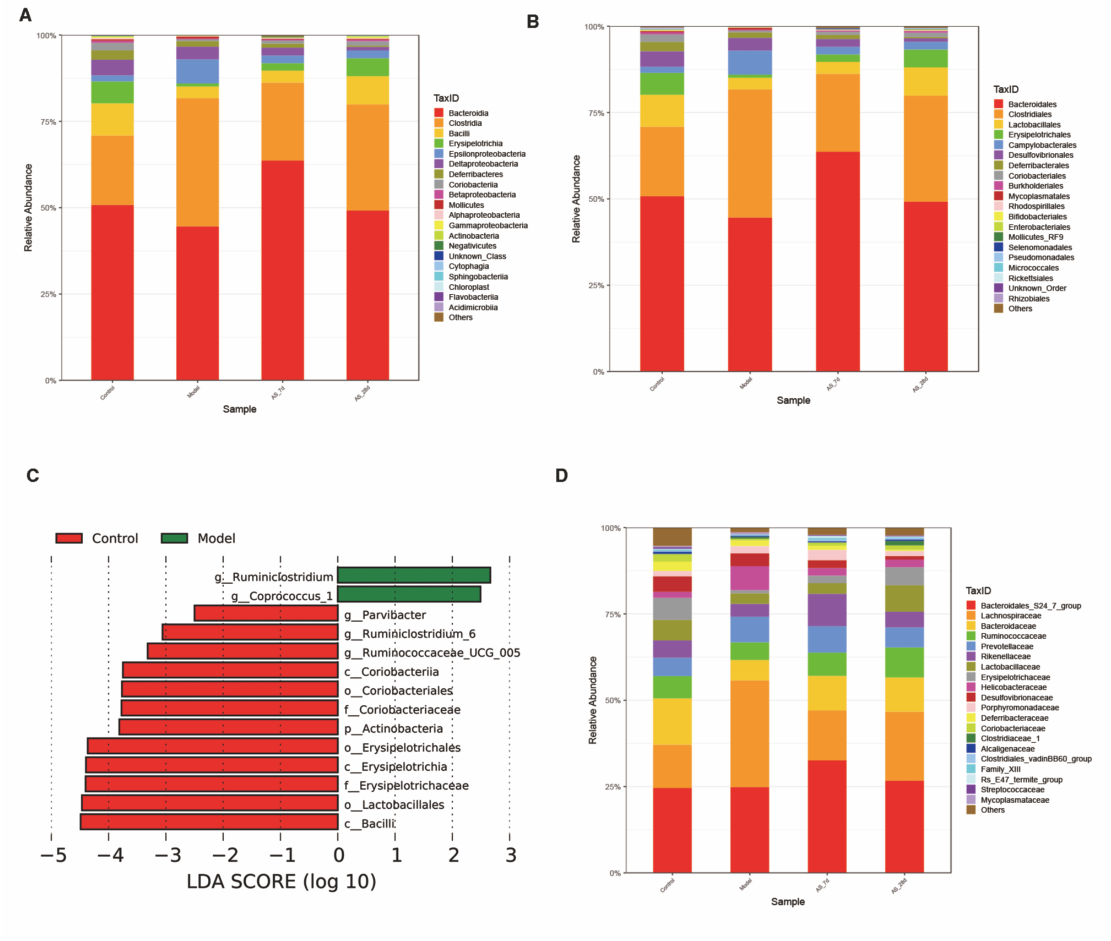


**Figure S3. Effect of AS extract and radiation on the composition of gut microbiota.** (A). The relative abundance of bacteria at the class level. (B). The relative abundance of bacteria at the order level. (C). The relative abundance of bacteria at the family level. (D). Linear discriminant analysis (LDA) effect size showing the most differentially significant abundant taxa enriched in microbiota from the Control vs. Model.
